# Distinct components of cardiovascular health are linked with age-related differences in cognitive abilities

**DOI:** 10.1038/s41598-022-27252-1

**Published:** 2023-01-18

**Authors:** Deborah L. O. King, Richard N. Henson, Rogier Kievit, Noham Wolpe, Carol Brayne, Lorraine K. Tyler, James B. Rowe, Cam-CAN, Edward T. Bullmore, Andrew C. Calder, Rhodri Cusack, Tim Dalgleish, John Duncan, Fiona E. Matthews, William D. Marslen-Wilson, Meredith A. Shafto, Karen Campbell, Teresa Cheung, Simon Davis, Linda Geerligs, Anna McCarrey, Abdur Mustafa, Darren Price, David Samu, Jason R. Taylor, Matthias Treder, Janna van Belle, Nitin Williams, Daniel Mitchell, Simon Fisher, Else Eising, Ethan Knights, Lauren Bates, Tina Emery, Sharon Erzinçlioglu, Andrew Gadie, Sofia Gerbase, Stanimira Georgieva, Claire Hanley, Beth Parkin, David Troy, Tibor Auer, Marta Correia, Lu Gao, Emma Green, Rafael Henriques, Jodie Allen, Gillian Amery, Liana Amuntse, Anne Barcroft, Amanda Castle, Cheryl Dias, Jonathan Dowrick, Melissa Fair, Hayley Fisher, Anna Goulding, Adarsh Grewale, Geoff Hale, Andrew Hilton, Frances Johnson, Patricia Johnston, Thea Kavanagh-Williamson, Magdalena Kwasniewska, Alison McMinn, Kim Norman, Jessica Penrose, Fiona Roby, Diane Rowland, John Sargeant, Maggie Squire, Beth Stevens, Aldabra Stoddart, Cheryl Stone, Tracy Thompson, Ozlem Yazlik, Dan Barnes, Marie Dixon, Jaya Hillman, Joanne Mitchell, Laura Villis, Kamen A. Tsvetanov

**Affiliations:** 1 Department of Clinical Neurosciences, University of Cambridge, Cambridge, CB2 0SP UK; 2 Department of Psychology, Centre for Speech, Language and the Brain, University of Cambridge, Cambridge, CB23 6HT UK; 3 Department of Psychiatry, University of Cambridge, Cambridge, CB2 2QQ UK; 4 Medical Research Council Cognition and Brain Sciences Unit, Cambridge, CB2 7EF UK; 5 Cambridge Centre for Ageing and Neuroscience (Cam-CAN), University of Cambridge and MRC Cognition and Brain Sciences Unit, Cambridge, CB2 7EF UK; 6 Donders Research Institute for Brain, Cognition and Behaviour, Radboud University, 6525 AJ Nijmegen, The Netherlands; 7 Cambridge Public Health, Cambridge Public Health, University of Cambridge, Cambridge, CB2 0SR UK; 8 Department of Physical Therapy, The Stanley Steer School of Health Professions, Sackler Faculty of Medicine, Tel Aviv University, Tel Aviv, Israel

**Keywords:** Cognitive ageing, Cardiovascular biology, Risk factors

## Abstract

Cardiovascular ageing contributes to cognitive impairment. However, the unique and synergistic contributions of multiple cardiovascular factors to cognitive function remain unclear because they are often condensed into a single composite score or examined in isolation. We hypothesized that vascular risk factors, electrocardiographic features and blood pressure indices reveal multiple latent vascular factors, with independent contributions to cognition. In a population-based deep-phenotyping study (n = 708, age 18–88), path analysis revealed three latent vascular factors dissociating the autonomic nervous system response from two components of blood pressure. These three factors made unique and additive contributions to the variability in crystallized and fluid intelligence. The discrepancy in fluid relative to crystallized intelligence, indicative of cognitive decline, was associated with a latent vascular factor predominantly expressing pulse pressure. This suggests that higher pulse pressure is associated with cognitive decline from expected performance. The effect was stronger in older adults. Controlling pulse pressure may help to preserve cognition, particularly in older adults. Our findings highlight the need to better understand the multifactorial nature of vascular aging.

## Introduction

Life expectancy is increasing and the global population is ageing at an unprecedented rate. Identifying the factors that promote healthy cognitive ageing is therefore a public health priority^1,2^, recognised by the World Health Organisation’s global strategy for collaborative action on healthy ageing^3^. This includes identifying the risk and modifying factors for cognitive decline.

The second leading cause of cognitive decline in older people, after neurodegeneration, is vascular disease^4^, and vascular pathology is present in three-quarters of autopsies in older populations^5^. There may be a continuum between vascular pathology, dementia and Alzheimer’s Disease in the oldest old^6^. Vascular factors trigger a cascade of cellular and molecular damage that remodels cerebral vessels and tissue^7–12^. Vascular factors include total blood pressure^13,14^, pulse pressure^15–17^, heart rate variability^9,18–23^, and body-mass index^24–27^. Each factor may have different underlying causes and consequences for a spectrum of brain pathologies contributing to any degree of cognitive decline, ranging from subjective cognitive decline to dementia ^28^.

Ageing links vascular factors with cognitive decline, however the mechanisms underpinning this link are not well characterised. It is not established whether multiple vascular factors act synergistically through one shared biological pathway, or rather act independently with distinct—and possibly additive—effects on cognition. Vascular factors are often condensed into summary scores^29,30^, or considered in isolation from one another (e.g.^22^). This approach hinders understanding of age-related changes in cognition, since different vascular factors may have different and interacting effects^31–34^. Furthermore, interactions of vascular factors with age, in predicting cognition, could be non-linear, but only linear effects are normally tested (e.g.^35^). Recent research indicates multiple, independent vascular pathways that are relevant to brain health and cognitive ageing^10,35,36^. We propose that vascular ageing is better captured by multiple latent factors, and that these factors contribute differentially to age-related changes in cognitive abilities.

Fluid intelligence is a core cognitive ability, likely contributing to all cognitive tests^37^. It encompasses working memory and executive functions, and is most strongly indexed by tests of abstract problem-solving, such as the Cattell test^33,38,39^. Importantly, it declines rapidly with adult age^40,41^. It is often contrasted with crystallized intelligence, which represents acquired and general knowledge. In contrast to fluid intelligence, crystallized intelligence remains relatively stable throughout life^38^, with only a small decline in late life or in dementia^39,40^.

Though they are positively correlated across individuals^42,43^, the difference between crystallized and fluid intelligence—their “discrepancy”—has been suggested as a sensitive measure of decline arising from brain injury, neurodegeneration and ageing^44–52^. A large discrepancy score can indicate abnormal cognitive ageing^47,53^, likely reflecting disproportionate declines in fluid relative to crystallized intelligence. An advantage of using this discrepancy score is that it can function as a surrogate measure of longitudinal change in fluid intelligence, estimated from cross-sectional data. This is because individual differences in fluid intelligence are likely to reflect several age-invariant determinants (e.g. genetic, education) that have nothing to do with ageing. By adjusting fluid intelligence for crystallized intelligence, such individual differences are reduced, and hence the discrepancy score better approximates longitudinal decline. In other words, crystallized intelligence can be used to adjust an individual’s current fluid intelligence on the basis of their likely fluid intelligence when they were younger. However, it should be noted that this adjustment is based on several assumptions, namely that (1) measurement of fluid and crystallized intelligence is invariant to age, (2) the two are highly correlated in youth, and (3) crystallized measures do not change with age. We revisit these assumptions in the Discussion. More importantly, little is known about what determines the degree of discrepancy in healthy ageing, and here we test whether vascular factors are important such contributors.

To investigate the relationship between multiple vascular measures and the cognitive ability discrepancy, we used data from the Cambridge Centre for Ageing and Neuroscience (Cam-CAN; www.cam-can.org), with 708 adults, aged 18 to 88^54^. The vascular measures were body mass index (BMI), heart rate, heart rate variability (represented by both low and high frequencies of the electrocardiogram, ECG^20^), and blood pressure. BMI is associated with increased cardiovascular risk^55^ and with differences in cerebral structure^56^, while HR is associated with white matter health^35,57^. Heart rate variability is the interval between heart beats. It typically declines with age and is associated with decreased cerebral blood flow and changes to cerebral structure and function^20,58^. Instead of representing blood pressure through simple systolic and diastolic measures, better insight may be achieved by transforming it into its steady and pulsatile components^59–66^. Therefore, we report pulse pressure (difference between systolic and diastolic blood pressure) and total blood pressure (the sum of systolic and diastolic, to be orthogonal to pulse pressure). There is substantial evidence that pulse pressure plays an important role in brain and cognitive health^11^. The observed vascular variables were then modelled with exploratory factor analysis, with the expectation of three latent vascular factors based on previous work^36^. The observed cognitive measures were the four sub-scores on the Cattell test, believed to capture fluid intelligence, and the Spot-The-Word and Proverbs tests, believed to capture crystallized intelligence (see^54^ for details). A confirmatory factor analysis was used to define the two Latent Cognitive Factors (LCF) of fluid and crystallized intelligence. The subtraction of the participant loadings of the fluid LCF from those of the crystallized LCF produced the ability discrepancy score^47^. The relationships between the ability discrepancy, the three latent vascular factors and age, as well as their interactions, were then investigated with multiple linear regression. We examined interactions with sex and medication (binary regressors for each of a number of drugs relevant to cardiovascular and cognitive health)^67–69^. We adjusted for self-reported general health and for education level, which may capture other differences in fluid intelligence not represented by our measures of current crystallized intelligence^70^.

We predicted (i) that latent Vascular Factors associated with “good” cardiovascular health would decrease with age, while the cognitive ability discrepancy would increase with age; and (ii) that some latent Vascular Factors would associate with the ability discrepancy over and above age, and with a strength of association that changes with age, whereby ability discrepancy in older people would be more dependent on their latent vascular factors scores.

## Methods

### Participants

**Figure 1:**
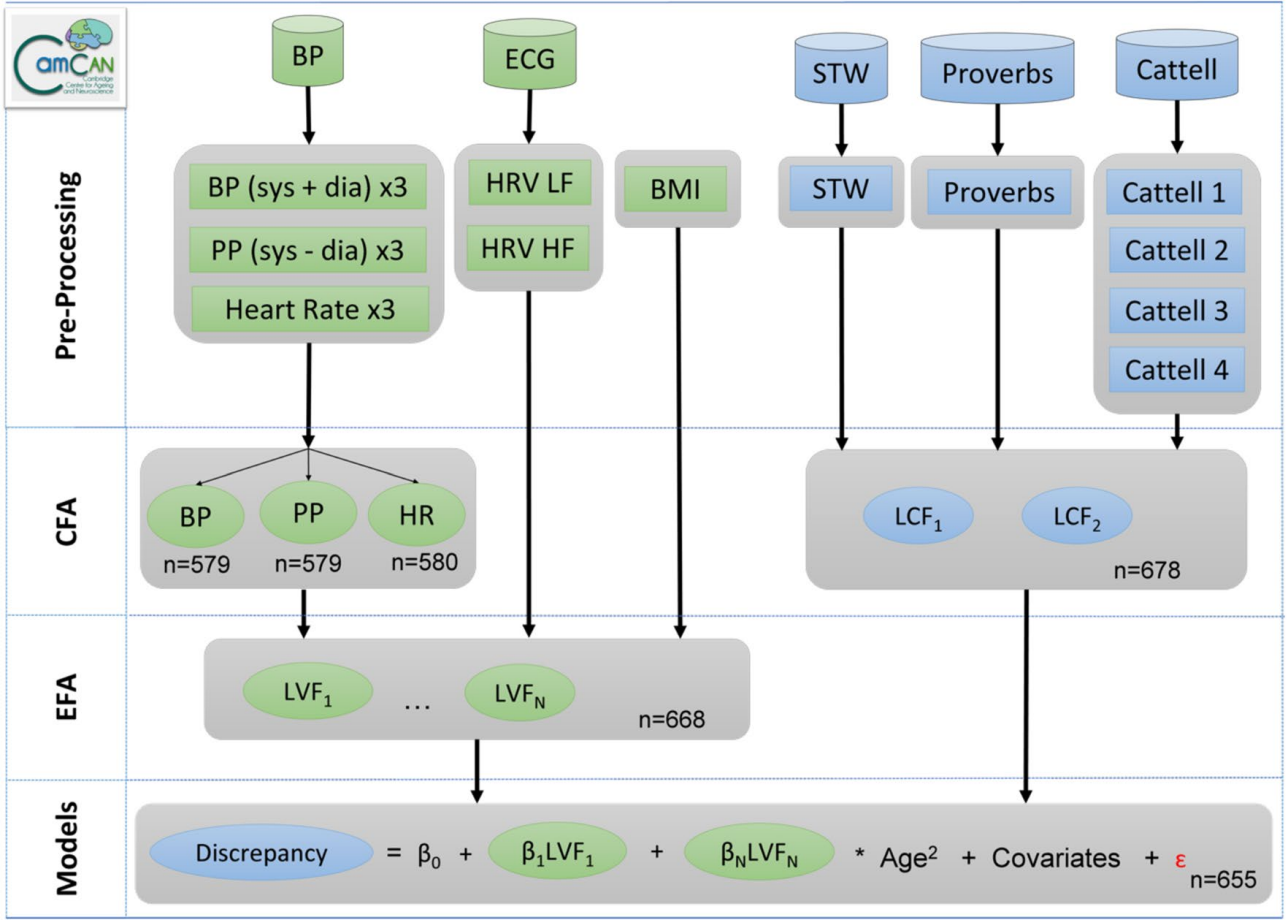
Schematic representation of the data processing and analysis pipeline to investigate shared and unique relationships between vascular and cognitive factors in the Cam-CAN dataset (n = 708). *BMI* body mass index, *BP* total blood pressure (systolic + diastolic), *dia* diastolic, *Cattell 1–4* sub-scores across the four Cattell tasks, *CFA* confirmatory factor analysis, Discrepancy, the ability discrepancy, defined as LCF2 (crystallized) minus LCF1 (fluid), *ECG* electrocardiogram, *EFA* exploratory factor analysis, *HR* heart rate, *HRV HF* heart rate variability at high frequency, *HRV LF* heart rate variability at low frequency, *LCF* latent cognitive factor, *LVF* latent vascular factor, *PP* pulse pressure (systolic − diastolic), *sys* systolic, *STW* spot the word.

Figure 1 illustrates the analytical strategy and the study design with the Cam-CAN cohort, n = 708^54,71^. The methods were carried out in accordance with guidelines approved by Cambridgeshire 2 (now East of England—Cambridge Central) Research Ethics Committee, who approved all experimental protocols. All participants gave full, informed, written consent. Participants were recruited from Cambridge City GPs, randomly selected from this complete population sampling frame. The detailed recruitment pathway is outlined in Supplementary Fig. 1. For a full list of exclusion criteria, see Supplementary Table 1. In brief, those participating represented the healthier and more advantaged spectrum within the population at all ages^54^. Self-reported general health was reported across four categories of: excellent, good, fair or poor. The diastolic and systolic blood pressure observations were excluded for one participant due to data entry errors. Education was reported across four categories of: none, GCSE or O-Level, A-Level, Degree (College or University). Medication status (binary on/off) was reported across four categories of drugs with cardiovascular relevance: [1] anti-hypertensives^69^, including angiotensin receptor blockers, angiotensin converting enzymes inhibitors, calcium channel blocking agents and thiazide diuretics; [2] beta blockers, including beta selective and non-selective beta blockers; [3] other diuretics, including loop and potassium sparing diuretics; [4] dyslipidemics drugs, including statins^67–69^.

### Vascular factors

Systolic and diastolic blood pressure, and heart rate, were measured using the A&D Medical Digital Blood Pressure Monitor (UA-774). Measurements were taken after at least 10 min of a participant being seated and repeated 3 times in succession. BMI was calculated using portable scales as weight (kg)/height (m)^2^. Heart rate variability was based on the frequency-domain information of normal-to-normal beats and extracted from resting state electrocardiogram recordings while seated during a separate MEG scan. We separated low- and high-frequency components: high frequency heart rate variability (0.15–0.4 Hz) principally indexes parasympathetic vagal influences, while low frequency heart rate variability (0.05–0.15 Hz) indexes non-vagal and sympathetic nervous system influences^20,72^. These two branches of the autonomic nervous system exhibit different non-linear trajectories with age, and might relate differently to cognition^73^. Heart rate variability data was processed using the PhysioNet Cardiovascular Signal Toolbox^74,75^ in MATLAB (Mathworks, MA). Following Tsvetanov et al.^36^, segments classified as atrial fibrillation were excluded and data for any participant with > 50% atrial fibrillation (n = 1) were excluded. The heart rate variability at low and high frequency, and BMI, were log-transformed to render them more Gaussian.

### Behavioural tasks

Crystallized intelligence was assessed through the Spot the Word and Proverb Comprehension tasks. In the Spot the Word test of vocabulary, participants were asked to point to the letter string in a pair that is a real word (albeit infrequent)^76^. In Proverb Comprehension, participants read and interpreted three English proverbs^77^. Fluid intelligence was assessed with the Cattell Culture Fair Test, Scale 2 Form A, in which participants completed non-verbal puzzles resulting in four summary scores based on series completion, classification, matrices and topology conditions^78,79^.

### Statistical analyses

Analyses were performed in R (version 4.0.2) and R-Studio^80^. For initial checks, the three observations of diastolic and systolic pressure were correlated with age, using the Pearson’s product moment correlation coefficient. They were then used to calculate three sets of total blood pressure (systolic + diastolic) and pulse pressure (systolic−diastolic). The resulting scores, and the three observations of heart rate, were log transformed to conform more closely to Gaussian distributions. These three sets of blood pressure, pulse pressure and heart rate measures were then standardised (mean = 0, standard deviation = 1) and condensed into a single latent variable per domain, using confirmatory factor analysis (CFA) in the lavaan package^81^. Latent variables reduce error and estimation bias, while increasing precision^82,83^. Note that the confirmatory models were saturated, where three indicators loading onto one latent variable gave zero degrees of freedom. Missing data were imputed using Full Information Maximum Likelihood in cases where data were recorded for at least one of the three domain-specific observations; where data were missing entirely, participants were omitted automatically (leaving n = 579 for blood pressure and pulse pressure; n = 580 for heart rate).

Next, we sought to identify the optimal number of factors among all vascular variables using Exploratory Factor Analyses (EFA). EFA is a common multivariate statistical method used to uncover the structure of a large set of variables^84^. Thus, EFA was used to identify the smallest number of latent vascular factors (latent vascular factors) that can parsimoniously explain the covariance observed among all vascular variables (blood pressure, n = 579; pulse pressure, n = 579; heart rate, n = 580; heart rate variability, at low and high frequencies, n = 604; BMI, n = 587). EFA was performed with the Psych package and imputing missing data (n = 668)^85^. Allowing two variables per latent factor is the upper boundary limit for model identification, meaning that one-, two- and three-factor solutions can be explored for six vascular variables in the current study. Note that a three-factor model with 6 variables will be fully saturated, not allowing estimation of the absolute fit indices. Therefore, model validity was based on model comparisons using the chi-squared statistic (*p* < 0.05), i.e. using comparative fit indices to determine the optimal number of latent vascular factors. Factor score estimates for each latent vascular factor were then extracted from the winning model for further regression analyses, below.

To explore the robustness of the winning EFA vascular model (and its loadings), we performed an additional structural equation model that included cognitive variables too (see Supplementary Section A). To additionally investigate whether the EFA vascular model structure is influenced by age, we repeated the EFA and model comparisons on sub-groups of young (n = 158, 18–37 years), middle (n = 311, 38–67 years) and old (n = 199, 68–88 years) participants (Table 1; Supplementary Section B).

Observed cognitive variables were standardised and condensed into two latent variables, using confirmatory factor analysis. The two-factor structure was based on the established dissociation between crystallized and fluid intelligence^86^. Scores on the Proverbs (n = 655) and Spot the Word tests (n = 705) loaded onto one latent cognitive factor (LCF1), representing crystallized intelligence. Scores on the Cattell tests (n = 660) loaded onto LCF2, representing fluid intelligence. Missing data were imputed using Full Information Maximum Likelihood in cases where data were recorded for at least one observed variable, producing LCFs for n = 678. The difference between LCF1 and LCF2 was calculated to give the ability discrepancy^47^. The calculation of the ability discrepancy was based on three assumptions, namely that (1) measurement of fluid and crystallized intelligence is invariant to age, (2) the two are highly correlated in youth, and (3) crystallized measures do not change with age. We investigated these assumptions using moderated non-linear factor analysis, correlations and visualisations, in Supplementary Section C.

The latent vascular factors and ability discrepancy were standardised to allow interpretation in terms of standard deviations from the mean. Linear and quadratic age predictor terms were also standardised. The relationships between the latent vascular factors, ability discrepancy and age were examined in multiple linear regression, using complete case analysis (n = 655). The presence of outliers with undue influence, as identified with Cook’s criteria^87^, motivated the use of robust linear regression, implemented in the MASS package^88^. We performed a series of regression models from simple to complex, all including general health, sex and education as covariates of no interest, but dropping effects that did not improve overall model fit. Fit was investigated with the Akaike Information Criterion, Bayesian Information Criterion and Sum of Squares. Results were reported at *p* < 0.05. To guide the interpretation of significance of parameters in the larger models, model specific p-values after Bonferroni corrections are also reported.

Five models were used to test different hypotheses. For model syntax, see Supplementary Section D. The first model examined the relationship between the ability discrepancy and the three latent vascular factors, ignoring any shared dependence on age, to reveal which latent vascular factor(s) make unique contributions to the ability discrepancy. The Second model investigated whether any relationships between the ability discrepancy and latent vascular factors remained over and above a second-order polynomial expansion of age, and/or whether any effects of latent vascular factors depended on age. Note that, since the latent vascular factors were highly correlated with age, if effects of latent vascular factors from Model 1 are no longer significant in Model 2, then this could simply be because age shares variability with the latent vascular factors. General health was covaried in Models 1 and 2, and taken forwards into further models if it significantly predicted the ability discrepancy.

Models 1 and 2 were compared and if Model 2 was shown to better fit the data, then the age terms were taken forwards into further models. Model 3 investigated whether our findings could be explained by medication status. Model 4 accounted for the interacting effects of sex on Vascular factors with age^9,89–91^. Since model comparisons showed that medications did not improve overall fit in Model 3, medications were not specified in Model 4. Model 5 investigated whether latent vascular factors interact with each other in order to determine the ability discrepancy. Since model comparisons showed that the inclusion of Sex interaction terms did not improve overall fit in Model 4, these interactions were not perpetuated to Model 5.

We also investigated whether the relationships between latent vascular factors and the ability discrepancy score, as explored in regression models, were robust to the effects of age on observed vascular measures. The EFA on vascular health was repeated over sub-groups of young, middle and old participants, and the resulting latent vascular factors were input to regression Models 1–5 (Supplementary Section B).

## Results

### Participants

Characteristics of the 708 participants in the Cam-CAN Phase 2 are outlined in Table 1. Rate of missing data varied between 0 and 18% (see Table 1). When the cohort (n = 708) was split into three age groups, the proportion of each on medications was: 0% of the younger (18–37 years); 12.6% of the middle (38–67 years) and 51.6% of the older (68–88 years) group. Across the entire cohort, 6.5% of participants had no education beyond 15 years; 13.6% had GCSEs or O-levels (usually taken at 16 years old); 19.5% had A-levels (usually taken at 18 years old); and 60.2% had a degree (or equivalent higher education). Also, across the entire cohort, 29.1% of participants rated their general health as excellent; 55.6% as good; 13.4% as fair; and 1.6% as poor.

**Table 1:** Demographic information by age-tertiles (n = 708).

	Complete data (n)			Missing (%)			Range / Number (Male)			Mean			SD		
	Young	Middle	Old	Young	Middle	Old	Young	Middle	Old	Young	Middle	Old	Young	Middle	Old
Age (years)	164	325	219	0	0	0	18–37	38–67	68–99	29.5	52.5	76.6	5.5	8.5	5.4
Sex (Male)	164	325	219	0	0	0	79	159	111	–	–	–	–	–	-
Diastolic (mmHG)	142	265	169	13.4	18.5	22.8	51.7–94.3	50–118.7	49–114.3	69.9	74.9	72.4	8.7	10.4	10.7
Systolic (mmHG)	142	265	169	13.4	18.5	22.8	92.3–141	79.3–172.3	82.3–178.3	112.3	118.7	130.1	11	14.6	18.9
Heart Rate (beats/minute)	142	265	169	13.4	18.5	22.8	43.3–95.7	39–96.3	44.7–107.7	65.5	64.5	68	10.1	9.5	12
HRV low frequency (ms^2^)	135	299	170	17.7	8	22.4	5.5–9283.7	13–15,784.9	1.3–1304.4	1163	834.9	231.8	1184.3	1393.2	257.7
HRV high frequency (ms^2^)	135	299	170	17.7	8	22.4	23.4–7129.8	10.2–5332.9	5.5–3543.8	1422.3	648.2	264.6	1226.6	767.3	378.1
BMI (kg/m^2^)	142	268	177	13.4	17.5	19.2	16.8–37.7	17.6–48.3	19.9–44.3	23.8	25.9	27.1	4	4.9	4
Cattell sub-score 1	154	307	199	6.1	5.5	9.1	6–12	3–12	2–12	10.6	9.8	7.8	1.3	1.7	2.1
Cattell sub-score 2	154	307	199	6.1	5.5	9.1	3–13	3–13	1–12	9.6	8.3	6.7	1.9	1.9	1.9
Cattell sub-score 3	154	307	199	6.1	5.5	9.1	7–12	4–12	1–12	10.6	9.5	7.2	1.3	1.7	2
Cattell sub-score 4	154	307	199	6.1	5.5	9.1	1 to 8	1 to 9	1 to 8	6.4	5.4	4.1	1.4	1.6	1.9
Proverbs	149	310	196	9.1	4.6	10.5	0–6	0–6	0–6	4.1	4.7	4.6	1.7	1.6	1.7
Spot the Word	163	325	217	0.6	0	0.9	24–60	30–60	29–60	51.4	54.2	54.3	5.8	4.5	5.9
Medications (percentage of total per drug)	–	–	–	0	0	0	–	–	–	–	–	–	–	–	-
Anti-hypertensives	0.0	8.0	37.4	–	–	–	–	–	–	–	–	–	–	–	-
Beta Blockers	0.0	0.9	9.6	–	–	–	–	–	–	–	–	–	–	–	-
Other diuretics	0.0	2.8	13.2	–	–	–	–	–	–	–	–	–	–	–	-
Dyslipidemics	0.0	8.6	28.3	–	–	–	–	–	–	–	–	–	–	–	-
Education (percentage of total by category)	–	–	–	0.6	0.0	0.5	–	–	–	–	–	–	–	–	-
No qualifications tried (< 16)	0.6	3.1	16.0	–	–	–	–	–	–	–	–	–	–	–	-
GCSEs / O-levels (age 16)	11.0	14.2	14.6	–	–	–	–	–	–	–	–	–	–	–	-
A-levels (age 18)	15.2	18.5	24.2	–	–	–	–	–	–	–	–	–	–	–	-
Degree (over 18)	72.6	64.3	44.7	–	–	–	–	–	–	–	–	–	–	–	-
General Health (percentage of total by category)	–	–	–	0.6	0	0.5	–	–	–	–	–	–	–	–	-
Excellent	18.9	34.8	28.3	–	–	–	–	–	–	–	–	–	–	–	-
Good	62.2	48.3	61.6	–	–	–	–	–	–	–	–	–	–	–	-
Fair	15.9	14.8	9.6	–	–	–	–	–	–	–	–	–	–	–	-
Poor	2.4	2.2	0	–	–	–	–	–	–	–	–	–	–	–	-

For the three observations of systolic, diastolic and heart rate, average values are reported. Measures presented here were averaged over the three observations. One decimal place is reported where data are continuous.

*BMI* body mass index, *GCSE* The General Certificate of Secondary Education, *HRV* heart rate variability, *SD* standard deviation.

### Vascular factors and age

Diastolic and systolic blood pressure increased with age (Supplementary Fig. 6). Within each confirmatory factor analysis on the repeated measures of blood pressure, heart rate and pulse pressure, there were positive associations between all observed and latent variables, with *p* < 0.001 for all factor loadings. The resulting latent variables for blood pressure, heart rate and pulse pressure showed significant positive associations with age (Supplementary Fig. 7). BMI also correlated significantly positively with age, while heart rate variability at both frequencies showed a significantly negative association (Supplementary Fig. 7).

### Vascular factor analysis

We used EFA to estimate models with one, two and three factors. The two-factor model did not converge well. The three-factor model was fully saturated (no residual degrees of freedom). Model comparisons showed two factors to fit better than one (∆ *X*^2^ = 236.57, *p* < 0.001), and three factors to fit better than two factors (∆ *X*^*2*^ = 95.04, *p* < 0.001). We confirmed the validity of the three-factor model in combination with cognitive measurements (see Supplementary Section A).

**Figure 2:**
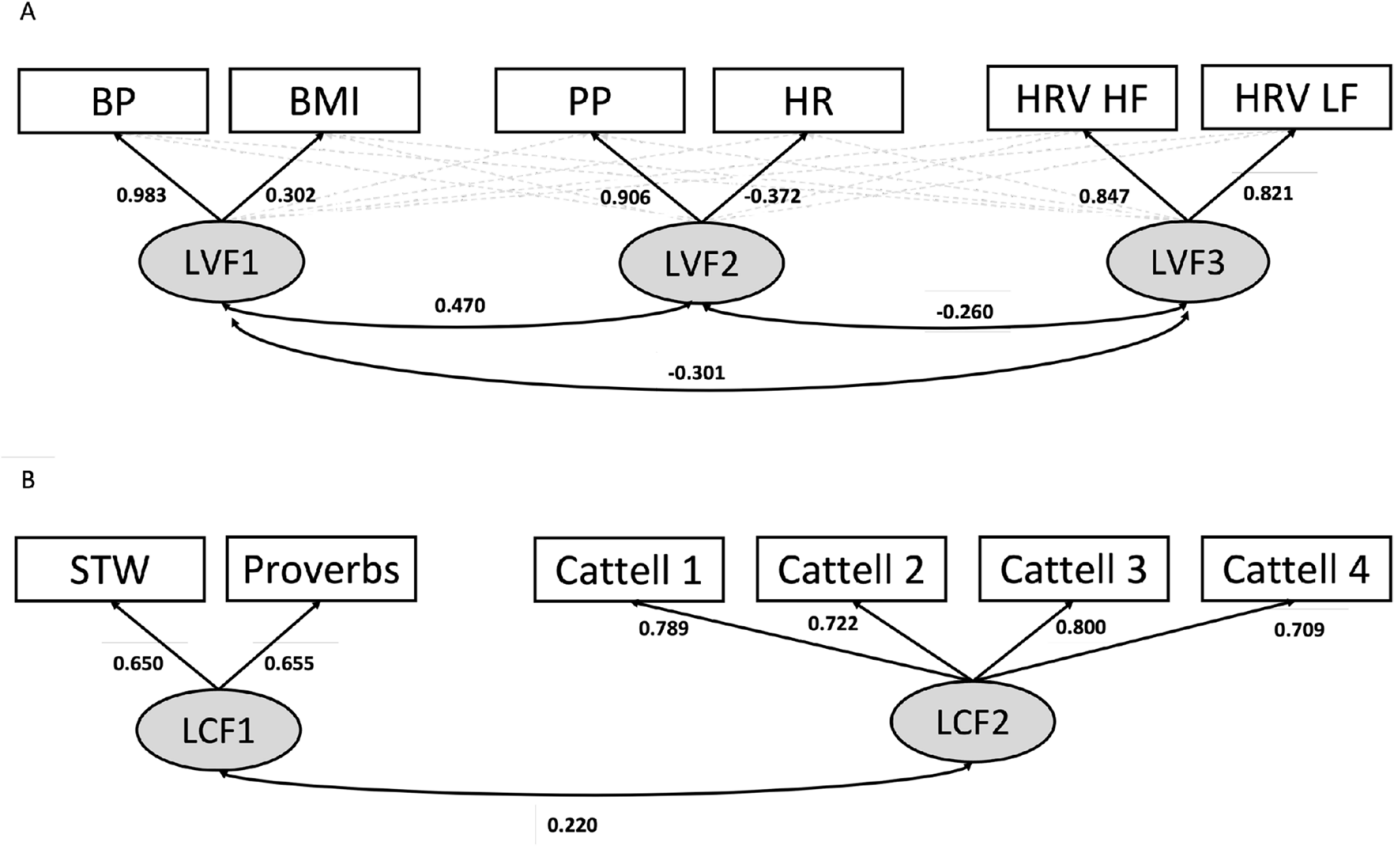
**A** The three-factor Exploratory Factor Analysis model of vascular health. The numeric values of cross-loadings < 0.30 (dashed grey arrows) are omitted here for visual clarity and reported fully in Supplementary Table 5. **B** The two-factor Confirmatory Factor Analysis model of cognition. *BMI* body mass index, *BP* total blood pressure, *HR* heart rate, *HRV HF* heart rate variability at high frequency, *HRV LF* heart rate variability at low frequency, *LCF* latent cognitive factor, *LVF* latent vascular factor, *PP* pulse pressure, *STW* spot the word.

**Figure 3:**
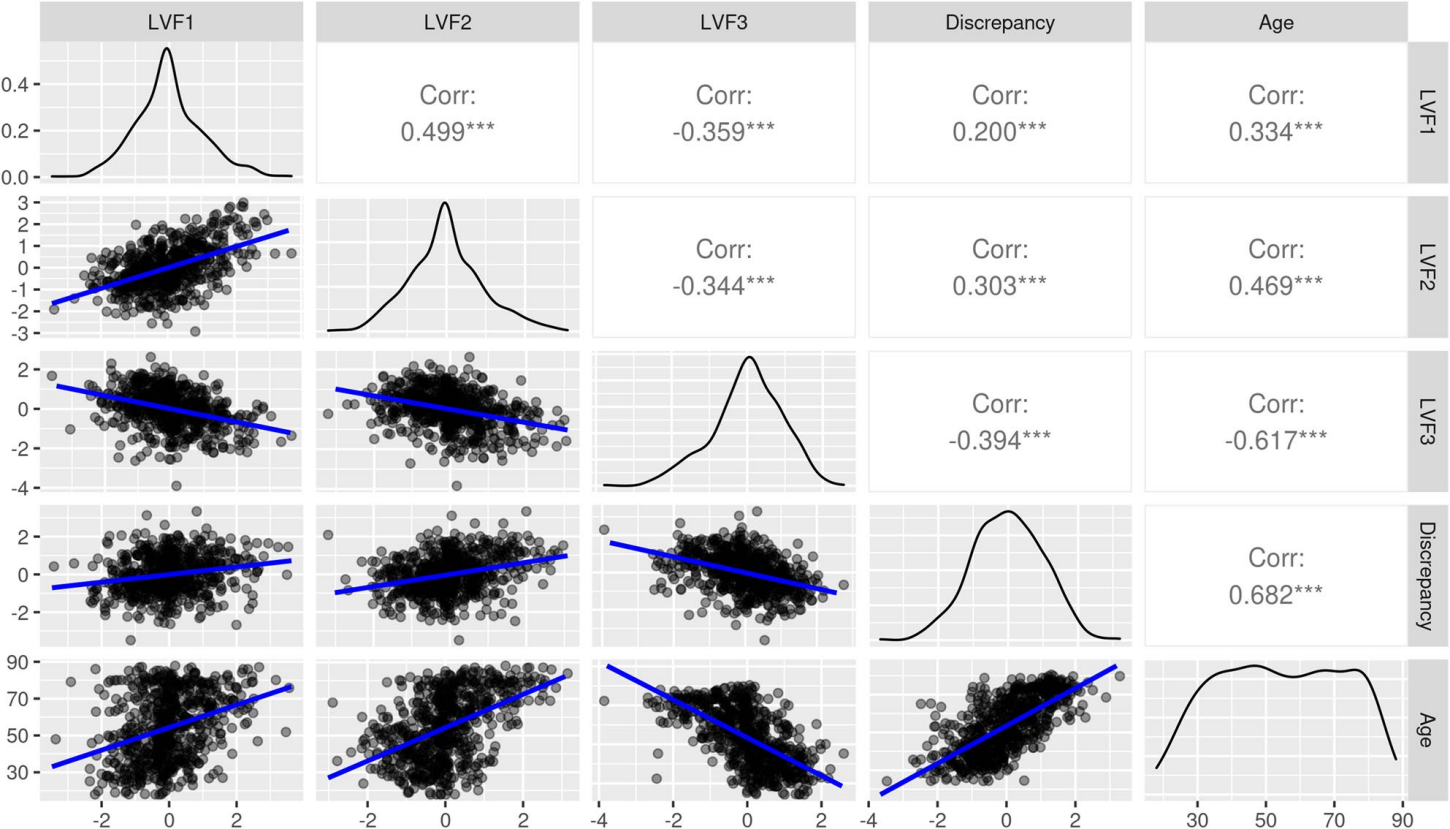
Scatter plots (lower left), distributions (leading diagonal) and Pearson correlations (upper right) for latent vascular factors, ability discrepancy and age. Scatter plots show linear associations (blue) and data intensity (greyscale). Stars indicate increasing significance on the correlations: ***, *p* < 0.001; **, *p* < 0.01; *, *p* < 0.05. *Corr* correlation coefficient, *Discrepancy* ability discrepancy, *LVF1-3* latent vascular factors 1–3.

The estimates of factor loadings and covariances for the EFA vascular model are visualised in Fig. 2A and detailed fully in Supplementary Table 5. Blood pressure loaded strongly onto the first latent factor, with a small contribution from BMI. Pulse pressure loaded strongly onto the second latent factor, with a small negative contribution from heart rate. Both frequencies of heart rate variability loaded similarly onto the third latent factor. All latent vascular factors correlated significantly with age (Fig. 3).

To explore whether the model structure remained consistent with age, we repeated EFA on three sub-groups of young, middle and old aged participants (Supplementary Section B). In all age groups, the three-factor model consistently fit best (Supplementary Table 2). Latent vascular factors produced in the age group specific and whole sample EFA correlated highly (r > 0.71, *p* < 0.001) (Supplementary Fig. 4).

### Ability discrepancy

The cognitive variables (Supplementary Fig. 8) were entered into a two-factor CFA and produced latent cognitive factor 1, representing crystallized intelligence, and latent cognitive factor 2, representing fluid intelligence (Fig. 2B). The ability discrepancy was calculated by subtracting the participant loadings on the fluid factor from those on the crystallized factor^47^. The calculation of the ability discrepancy was based on three assumptions, firstly that the measurement of fluid and crystallized intelligence is invariant to age. Moderated non-linear factor analysis suggested that the measurement model did not differ substantially across the continuous covariate of age, warranting us to use the factor scores across the lifespan (Supplementary Section C). On the second and third additional assumptions, crystallized and fluid intelligence correlated highly in young adults, and crystallized intelligence remained stable with age (Supplementary Section C). The ability discrepancy showed a strong positive association with age (Fig. 3). It also correlated significantly with the three latent vascular factors, with substantial effect sizes (Fig. 3).

### Multiple linear regression

In Model 1 (n = 655, DoF = 642, residual standard error = 0.82), ability discrepancy showed a significant positive relationship with latent vascular factor 2 (std β = 0.195, SE = 0.043, *p* < 0.001) and a significant negative relationship with latent vascular factor 3 (std β = -0.347, SE = 0.039, *p* < 0.001) (Supplementary Table 6). Thus, while all three latent vascular factors explained shared variance, latent vascular factors 2 and 3, but not 1, made unique contributions to the ability discrepancy.

**Figure 4:**
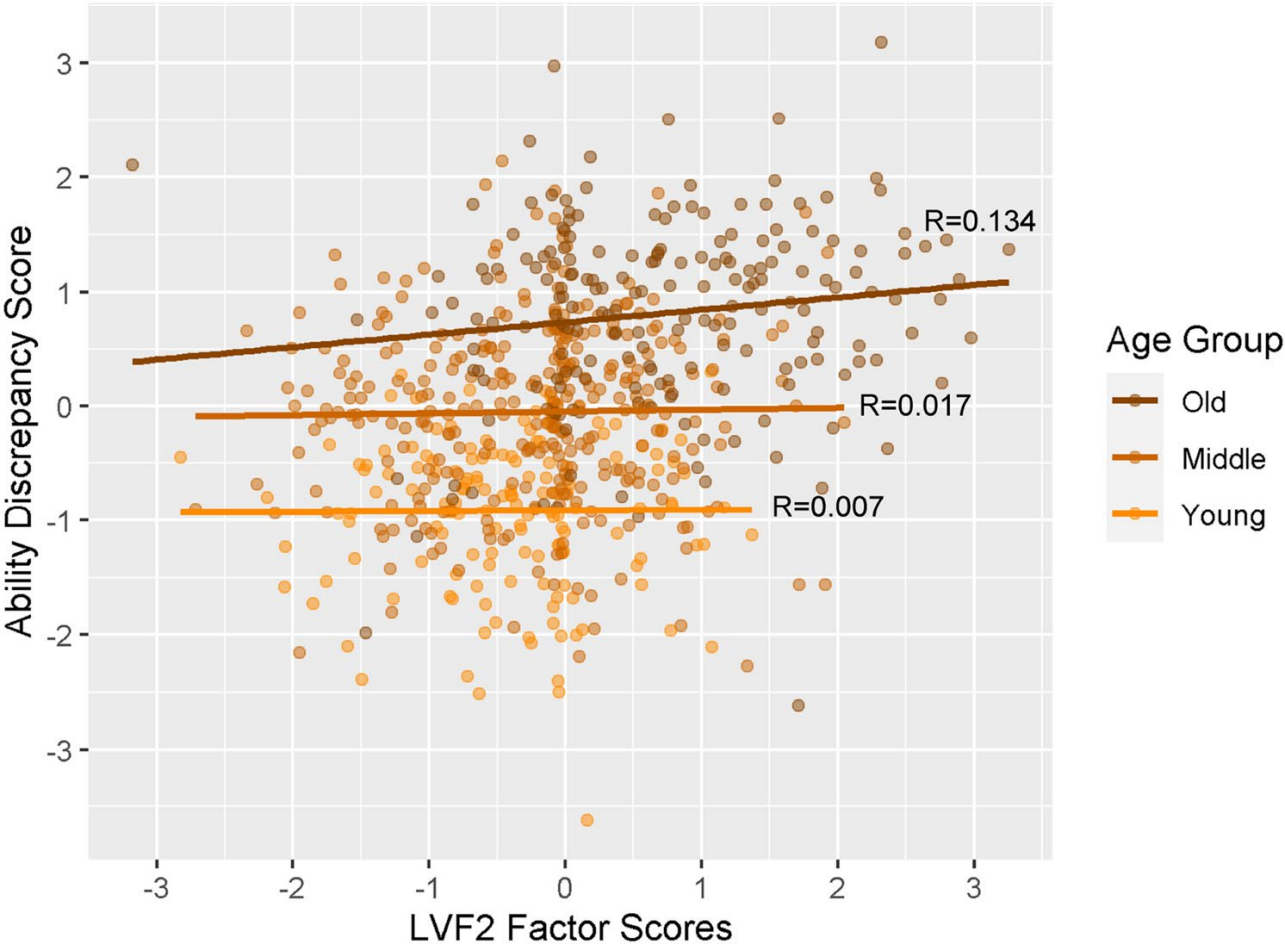
A visualisation of the effect of latent vascular factor 2, expressing predominantly pulse pressure, on the ability discrepancy for complete case data (n = 655). Note that age was a continuous variable for the interaction tested, but here participants are plotted as discrete groups of young (18–37 years, n = 154), middle (38–67 years, n = 307) and old (68–88 years, n = 194) for visualisation purposes only. *LVF2* latent vascular factor 2.

Compared to Model 1, Model 2 (n = 655, DoF = 634, residual standard error = 0.65) fit the data better (Supplementary Table 7). Model 2 revealed that the main effects of latent vascular factors 2 and 3 did not remain significant when accounting for age. The linear effect of age was significant, std β = 0.711, SE = 0.047, *p* < 0.001. More interesting was a significant interaction between latent vascular factor 2 and the quadratic effects of age (std β = 0.080, SE = 0.037, *p* = 0.030). This interaction is visualised in Fig. 4, by splitting the data into three age groups. It can be seen that the positive relationship between latent vascular factor 2 and the ability discrepancy is over 7 times stronger in the older group compared with the two younger groups.

From Model 2, there was no significant improvement in fit when adding medications (Model 3), interactions with sex (Model 4), or interactions between latent vascular factors (Model 5) (Supplementary Table 8). Given the lack of evidence supporting these more complex models, any significant parameters in Models 3–5 (Supplementary Tables 9–11) should be considered as suggestive only, and may not survive correction for multiple comparisons.

## Discussion

In this study, we show that changes in vascular systems across the lifespan have multifactorial effects on cognitive function. There are three key observations. First, we identify three latent vascular factors that broadly dissociate the autonomic nervous system from distinct components of blood pressure. Second, these factors make distinct contributions to age-related cognitive decline, as indexed by the “ability discrepancy” score^47^. Third, the pulse pressure factor was particularly associated with the cognitive ability discrepancy, increasingly so for older adults. This remained even after controlling for the use of hypertensive medications and the covariates of sex, education and general health. Importantly, the effect of pulse pressure was independent of other latent vascular factors. We propose that steps to maintain lower pulse pressure may help to preserve cognitive function into old age.

### Three components of vascular health

A single composite measure was insufficient to capture vascular health in our exploratory analyses, and the model fit was better with three factors. The evidence for these multiple latent vascular factors is consistent with previous model-based and data-driven approaches^35,36,92–95^. There was no evidence that the number of factors changed with age, at least in the sense of fewer factors being needed for optimal fit in young, middle or older age sub-groups. The three latent vascular factors were composed predominantly of two major blood pressure variables and an autonomic nervous system variable; a decomposition that agrees with previous studies^36,95^. This decomposition also mimics established models of cardiovascular health (discussed below). The unsupervised construction of these latent vascular factors highlights their distinct contributions, which may involve different pathways and require different interventions.

The first factor, latent vascular factor 1, expressed total blood pressure, which is the steady state component of blood pressure (Fig. 2). This component is proposed to be mainly influenced by cardiac output and peripheral vascular resistance^64^. The additional contribution of BMI to latent vascular factor 1 fits well with early work showing a strong correlation between BMI and the steady component of blood pressure^59^. Latent vascular factor 1 was positively associated with age and the ability discrepancy (Fig. 3), however it did not significantly predict the ability discrepancy, over and above other latent vascular factors, in Model 1 (Supplementary Table 4). Consistent with these observations, Lefferts et al.^62^ showed that steady blood pressure no longer predicts cognition over and above the effects of pulsatile blood pressure and covariates. We previously found that the steady component of blood pressure is associated with age-related cerebrovascular dysfunction of the sensorimotor regions, independently of the pulsatile component^36^. This suggests a unique contribution of steady blood pressure to brain health, with regional specificity. Future work should establish the possibility of a specific contribution of steady state blood pressure to brain functioning, and whether this varies across the lifespan^96^.

The second component, latent vascular factor 2, expressed pulse pressure, with some contribution from heart rate (Fig. 2). Latent vascular factor 2 was positively associated with age (Fig. 3). Given the predominant loading by pulse pressure, latent vascular factor 2 likely represents a cerebrovascular element^97,98^. This is also consistent with previous findings that pulse pressure and white matter lesion burden expressed a common latent cerebrovascular factor^36^.

Latent vascular factor 3 expressed resting heat rate variability (Fig. 2), which indexes a specific component of the autonomic nervous system^22,99,100^. The decomposition of autonomic nervous system signals separately from cerebrovascular health signals (latent vascular factors 1 and 2) confirms that these are distinct, yet partly correlated, constructs of vascular ageing^101^. The convergence of low and high frequencies of resting heart rate variability onto latent vascular factor 3 does not necessarily rule out frequency-specific effects on cognition, or frequency-specific effects of task-based heart rate variability modulation/reactivity^102,103^. Latent vascular factor 3 associated negatively with age, consistent with previous studies on heart rate variability.

### Pulse pressure and age-related cognitive function

A novel aspect of our work was to simultaneously relate the three latent vascular factors to age-related differences in cognition, specifically the cognitive discrepancy score. In the absence of longitudinal data, this discrepancy is arguably (see below) a better estimate age-related change than raw individual differences in fluid intelligence. When relating directly to cognitive ability, only latent vascular factors 2 and 3 made unique contributions. Note that this does not mean latent vascular factor 1 has no relationship with cognitive ability; only that we cannot distinguish any such contribution from those of factors 2 and 3. The negative relationship for latent vascular factor 3 shows that higher heart-rate variability is associated with lower ability discrepancy, i.e., more variable heart rate is associated with less discrepancy, i.e., fluid intelligence that is closer to what would be expected from crystallized intelligence.

However, the relationship between latent vascular factor 3 and cognitive discrepancy was no longer significant when adjusting for age. This is consistent with cross-sectional studies where the association between resting heart rate variability and executive functions is accounted for by age and systemic vascular health^104–106^. Future studies should investigate how the shared variance between heart rate variability, systemic vascular health, age and cognition is linked to changes in cerebral blood flow, tissue integrity and neural function^9,20,58^. Heart rate variability is also theorised to link to domain specific measures of cognition, including emotional regulation^20–22,91,107^. Future research should also explore whether heart rate variability affects emotional regulation independently of other vascular factors, and whether this relationship changes with age.

Latent vascular factor 2 made a unique, positive contribution to the ability discrepancy, consistent with higher pulse pressure being detrimental to cognitive ability. Like latent vascular factor 3, this contribution was no longer significant when adding age to the model. This could be because age is the true driver of ability discrepancy, or that age and latent vascular factor 2 are so highly correlated that we can no longer detect a unique effect of the latter. More importantly, we did find a significant interaction between age and latent vascular factor 2. This was a quadratic effect, consistent with pulse pressure being especially important for cognition in old age, rather than changing linearly with age. These findings are consistent with a growing body of literature suggesting that pulsatile, rather than steady, blood pressure is an important factor for brain health and higher cognitive functions^15,16,62,108–110^. Future work needs to evaluate whether maintaining normal pulse pressure across the lifespan is the mediating factor of cognitive function and plays a role in the increasing relationship between brain function and cognition in old age^111–115^.

The mechanism by which pulse pressure relates to the ability discrepancy has yet to be identified. Pulse pressure has been proposed to be associated with a trigger point of a positive feedback loop of rising arterial stiffness and pressure that penetrates increasingly into deep brain tissue^11^. This causes a cascade of molecular and cellular damage to cerebral vessels, which ultimately injures the blood brain barrier, promoting the aggregation of beta-amyloid^116–118^, phosphorylated tau^119^ and white matter hyperintensities^11,97,98,120^. Pulse pressure induced hippocampal damage is theorised to result in impaired episodic memory^98^, while it also uniquely contributes to cerebrovascular dysfunction in frontoparietal regions^36^. The recruitment of frontoparietal regions is of particular interest here, given that their involvement in fluid ability processing is partly explained by age-related hypoperfusion in these areas^121^. Separately, pulse pressure is associated with amyloid-dependent hypometabolism in frontoparietal regions^122^, which has in turn been associated with the ability discrepancy score^47^. Our research adds to this evidence by showing, in a population-based lifespan cohort, that pulsatile, rather than steady state blood pressure, is a predictor of cognitive decline (ability discrepancy), and this is particularly so for older adults. We propose that pulse pressure links to cognitive ageing through a distinct mechanism of cellular and molecular changes in cerebral vessels^36,98,121^. We further highlight pulse pressure as an emerging therapeutic target to prevent cognitive decline in ageing^97^.

### Limitations

Our study has limitations. The results were based on a population-based cross-sectional cohort and cannot directly speak to longitudinal ageing, i.e. individuals’ progression over time. For example, cross-sectional data may also be confounded by generational effects, such as the general increase in education and a decrease in blood pressure seen across recent decades. While we tried to approximate cognitive change via the discrepancy score (see below), our conclusions are restricted to the effects of age and its correlates, as assessed across individuals, and we cannot rule out the possibility that differences in vascular health are the consequence rather than the cause of differences in cognitive ability. Nonetheless, though the cross-sectional nature of our study cannot speak directly to the expansive body of literature on effects of mid-life blood pressure on late-life cognition^109,123,124^, our findings can generate hypotheses to test in longitudinal datasets. It should also be noted that the population-based adult-lifespan sample (18–88 years) used here is likely to be healthier, with lower variability in cardiovascular function, than samples used in other reports^54^.

The discrepancy between an individual’s score on fluid versus crystallized intelligence was used to approximate cognitive decline, on the assumptions that 1) measurement of fluid and crystallized intelligence is invariant to age, 2) the two are highly correlated in youth, and 3) crystallized measures do not change with age. Each of these assumptions was tested and satisfied (see Supplementary Section C). In brief, we showed that the measurement model for fluid and crystallized intelligence was invariant to age. Secondly, though crystallized intelligence showed some increase from youth to mid-life (which could be generational), and though it has previously been shown to decline in very old age^70^, any such effects of ageing were much smaller than those on fluid intelligence. Finally, the two measures were highly correlated in young adulthood. It is possible that this high correlation means that some aspects of general cognitive ability are lost when subtracting them, though the assumption here is that this shared variance largely reflects age-invariant individual differences (such as genetics), and so is less relevant to our present question about vascular effects on cognitive ageing. Indeed, it has been argued that accounting for crystallized abilities helps adjust scores on tests of fluid ability that may be artificially lower than expected, e.g. owing to verbal materials and/or complex instructions in such tests^125^.

We estimated a subset of potential vascular factors, based on a limited set of measures on a single visit. Our measures were relatively easy to acquire in practice on a large scale. The reactivity of the autonomic system to an event or stressor, which may be cognitive, emotional or physical in nature, e.g. phasic heart rate variability^102^, could prove more sensitive to the resting state heart rate variability estimates used here. It is also possible that there are more than three latent vascular factors, but we cannot test that here with six vascular measures^84^, and to do so would require a greater number of vascular measures to be collected. For example, future work could test whether cholesterol levels^126^ comprise an independent factor, or instead load on one or more of the three latent vascular factors identified here, or examine lifestyle factors such as physical activity, smoking and socio-economic status.

It may seem surprising that we found no evidence that medications related to vascular health contribute to ability discrepancy, or at least modulate the effects of our latent vascular factors on ability discrepancy. This may be because the indirect effects of medication on ability discrepancy are mediated fully through their direct effects on latent vascular factors. Alternatively, it could be that, while medications affect current latent vascular factors, they may be given too late to prevent pre-medication levels of vascular factors like pulse pressure from already causing irreversible effects on cognitive ability^127^, or that the effects of chronic stable medication are mitigated by homeostasis. Either way, future research could investigate the mechanisms through which latent vascular factors mediate cognitive change, for example through damage to cerebral vessels, changes in brain perfusion or neuroinflammation, perhaps by direct manipulation of medications.

In summary, we show that vascular ageing has multifactorial relationships with cognitive ageing. Of the three latent vascular factors, an increase in the factor expressing pulse pressure was uniquely associated with the cognitive discrepancy score, and this relationship was stronger for older adults. We suggest that maintaining low pulse pressure may help to preserve cognitive function into old age.

## Supplementary Information

Supplementary Information.

## Data Availability

The dataset is freely accessible on the Cam-CAN portal, subject to a data sharing agreement request: https://camcan-archive.mrc-cbu.cam.ac.uk/dataaccess. Analysis scripts can be downloaded: https://github.com/DebsKing/Distinct_Vascular_Components_Relate_To_Cognition.

## References

[1] Brayne C (2007). The elephant in the room—healthy brains in later life, epidemiology and public health. Nat. Rev. Neurosci..

[2] Deary IJ (2009). Age-associated cognitive decline. Br. Med. Bull..

[3] WHO | Dementia: a public health priority. *WHO* http://www.who.int/mental_health/publications/dementia_report_2012/en/.

[4] Matthews FE (2009). Epidemiological Pathology of Dementia: Attributable-Risks at Death in the Medical Research Council Cognitive Function and Ageing Study. PLOS Med..

[5] Neuropathology Group. Medical Research Council Cognitive Function and Aging Study. Pathological correlates of late-onset dementia in a multicentre, community-based population in England and Wales. Neuropathology Group of the Medical Research Council Cognitive Function and Ageing Study (MRC CFAS). *Lancet Lond. Engl.* **357**, 169–175 (2001). 10.1016/s0140-6736(00)03589-3 11213093

[6] Savva GM (2009). Age, Neuropathology, and Dementia. N. Engl. J. Med..

[7] Barnes JN (2015). Exercise, cognitive function, and aging. Adv. Physiol. Educ..

[8] de la Torre JC (2012). Cerebral hemodynamics and vascular risk factors: setting the stage for Alzheimer’s disease. J. Alzheimers Dis. JAD.

[9] Koenig J (2021). Cortical thickness and resting-state cardiac function across the lifespan: A cross-sectional pooled mega-analysis. Psychophysiology.

[10] Qiu C, Fratiglioni L (2015). A major role for cardiovascular burden in age-related cognitive decline. Nat. Rev. Cardiol..

[11] Thorin-Trescases N (2018). Impact of pulse pressure on cerebrovascular events leading to age-related cognitive decline. Am. J. Physiol. Heart Circ. Physiol..

[12] Zimmerman B, Rypma B, Gratton G, Fabiani M (2021). Age-related changes in cerebrovascular health and their effects on neural function and cognition: A comprehensive review. Psychophysiology.

[13] Jennings JR, Muldoon MF, Allen B, Ginty AT, Gianaros PJ (2021). Cerebrovascular function in hypertension: Does high blood pressure make you old?. Psychophysiology.

[14] Walker KA, Power MC, Gottesman RF (2017). Defining the relationship between hypertension, cognitive decline, and dementia: A review. Curr. Hypertens. Rep..

[15] Waldstein SR (2008). Pulse pressure and pulse wave velocity are related to cognitive decline in the baltimore longitudinal study of aging. Hypertension.

[16] Mitchell GF (2011). Arterial stiffness, pressure and flow pulsatility and brain structure and function: the Age, Gene/Environment Susceptibility—Reykjavik Study. Brain.

[17] Meyer ML (2017). Association of central arterial stiffness and pressure pulsatility with mild cognitive impairment and dementia: The atherosclerosis risk in communities study-neurocognitive study (ARIC-NCS). J. Alzheimers Dis..

[18] Appelhans BM, Luecken LJ (2006). Heart rate variability as an index of regulated emotional responding. Rev. Gen. Psychol..

[19] Geisler FCM, Vennewald N, Kubiak T, Weber H (2010). The impact of heart rate variability on subjective well-being is mediated by emotion regulation. Personal. Individ. Differ..

[20] Forte G, Favieri F, Casagrande M (2019). Heart rate variability and cognitive function: A systematic review. Front. Neurosci..

[21] Mather M, Thayer J (2018). How heart rate variability affects emotion regulation brain networks. Curr. Opin. Behav. Sci..

[22] Thayer JF, Hansen AL, Saus-Rose E, Johnsen BH (2009). Heart rate variability, prefrontal neural function, and cognitive performance: The neurovisceral integration perspective on self-regulation, adaptation, and health. Ann. Behav. Med..

[23] Varadhan R (2009). Frailty and impaired cardiac autonomic control: new insights from principal components aggregation of traditional heart rate variability indices. J. Gerontol. Ser. A.

[24] Albanese E (2017). Body mass index in midlife and dementia: Systematic review and meta-regression analysis of 589,649 men and women followed in longitudinal studies. Alzheimers Dement. Amst. Neth..

[25] Kivimäki M (2018). Body mass index and risk of dementia: Analysis of individual-level data from 1.3 million individuals. Alzheimers Dement. J. Alzheimers Assoc..

[26] Michaud TL (2018). The association between body mass index, and cognitive, functional, and behavioral declines for incident dementia. J. Alzheimers Dis. JAD.

[27] Veronese N (2017). Weight loss is associated with improvements in cognitive function among overweight and obese people: A systematic review and meta-analysis. Neurosci. Biobehav. Rev..

[28] van der Flier WM (2018). Vascular cognitive impairment. Nat. Rev. Dis. Primer.

[29] DeRight J, Jorgensen RS, Cabral MJ (2015). Composite cardiovascular risk scores and neuropsychological functioning: a meta-analytic review. Ann. Behav. Med. Publ. Soc. Behav. Med..

[30] Veldsman M (2020). Cerebrovascular risk factors impact frontoparietal network integrity and executive function in healthy ageing. Nat. Commun..

[31] Harada CN, Natelson Love MC, Triebel K (2013). Normal cognitive aging. Clin. Geriatr. Med..

[32] Horn JL, Cattell RB (1967). Age differences in fluid and crystallized intelligence. Acta Psychol. (Amst.).

[33] Shafto MA (2020). Cognitive diversity in a healthy aging cohort: cross-domain cognition in the cam-CAN project. J. Aging Health.

[34] Waldstein SR, Katzel LI (2015). Considering cumulative risk for cardiovascular disease in relation to cognitive function: a comment on DeRight et al.. Ann. Behav. Med. Publ. Soc. Behav. Med..

[35] Fuhrmann D (2019). Strong and specific associations between cardiovascular risk factors and white matter micro- and macrostructure in healthy aging. Neurobiol. Aging.

[36] Tsvetanov KA (2021). The effects of age on resting-state BOLD signal variability is explained by cardiovascular and cerebrovascular factors. Psychophysiology.

[37] Cattell RB (1963). Theory of fluid and crystallized intelligence: A critical experiment. J. Educ. Psychol..

[38] Manard M, Carabin D, Jaspar M, Collette F (2014). Age-related decline in cognitive control: the role of fluid intelligence and processing speed. BMC Neurosci..

[39] de Mooij SMM, Henson RNA, Waldorp LJ, Kievit RA (2018). age differentiation within gray matter, white matter, and between memory and white matter in an adult life span cohort. J. Neurosci..

[40] Salthouse TA (2009). When does age-related cognitive decline begin?. Neurobiol. Aging.

[41] Kievit RA (2016). A watershed model of individual differences in fluid intelligence. Neuropsychologia.

[42] Tucker-Drob EM (2022). A strong dependency between changes in fluid and crystallized abilities in human cognitive aging. Sci. Adv..

[43] Tucker-Drob EM, Brandmaier AM, Lindenberger U (2019). Coupled cognitive changes in adulthood: A meta-analysis. Psychol. Bull..

[44] Dierckx E (2008). Differentiation between dementia and depression among older persons: can the difference between actual and premorbid intelligence be useful?. J. Geriatr. Psychiatry Neurol..

[45] Harrington KD (2018). The effect of preclinical Alzheimer’s disease on age-related changes in intelligence in cognitively normal older adults. Intelligence.

[46] McCarthy F, Burns WJ, Sellers AH (2005). Discrepancies between premorbid and current IQ as a function of progressive mental deterioration. Percept. Mot. Skills.

[47] McDonough IM (2016). Discrepancies between fluid and crystallized ability in healthy adults: a behavioral marker of preclinical Alzheimer’s disease. Neurobiol. Aging.

[48] McDonough IM, Popp TE (2020). Linear and nonlinear relationships between cognitive subdomains of ability discrepancy and Alzheimer’s disease biomarkers. Neuropsychology.

[49] O’Carroll RE, Gilleard CJ (1986). Estimation of premorbid intelligence in dementia. Br. J. Clin. Psychol..

[50] O’Shea DM (2018). Discrepancies between crystallized and fluid ability are associated with frequency of social and physical engagement in community dwelling older adults. J. Clin. Exp. Neuropsychol..

[51] Rabbitt P (1993). Does it all go together when it goes? The Nineteenth Bartlett Memorial Lecture. Q. J. Exp. Psychol. A.

[52] Tomassini, A. *et al. Prefrontal cortical connectivity mediates locus coeruleus noradrenergic regulation of inhibitory control in older adults* (2021). 10.1101/2021.06.29.450427. 10.1523/JNEUROSCI.1361-21.2022 PMC9034774 35277392

[53] Bajpai S (2022). Discrepancy in fluid and crystallized intelligence: An early cognitive marker of dementia from the LASI-DAD cohort. Dement. Geriatr. Cogn. Disord. Extra.

[54] Shafto MA (2014). The Cambridge Centre for Ageing and Neuroscience (Cam-CAN) study protocol: A cross-sectional, lifespan, multidisciplinary examination of healthy cognitive ageing. BMC Neurol..

[55] Ndumele CE (2014). Obesity, subclinical myocardial injury, and incident heart failure. JACC Heart Fail..

[56] Ronan L (2016). Obesity associated with increased brain age from midlife. Neurobiol. Aging.

[57] Cox SR (2019). Associations between vascular risk factors and brain MRI indices in UK Biobank. Eur. Heart J..

[58] Shaffer F, Ginsberg JP (2017). An overview of heart rate variability metrics and norms. Front. Public Health.

[59] Darne B, Girerd X, Safar M, Cambien F, Guize L (1989). Pulsatile versus steady component of blood pressure: A cross-sectional analysis and a prospective analysis on cardiovascular mortality. Hypertens. Dallas Tex.

[60] Kim IB, Fealy N, Baldwin I, Bellomo R (2011). A pilot study of the epidemiology and associations of pulse pressure variation among non-cardiac surgery critically ill patients. Crit. Care Resusc. J. Australas Acad. Crit. Care Med..

[61] Laosiripisan J, Haley AP, Tanaka H (2017). Steady state versus pulsatile blood pressure component and regional cerebral perfusion. Am. J. Hypertens..

[62] Lefferts WK, Heffernan KS, Barreira TV (2016). Association between pulsatile blood pressure and cognitive performance among older adults: Insight from the National Health and Nutrition Examination Survey 1999–2002. Int. J. Cardiol..

[63] Roman MJ, Devereux RB (2014). Association of central and peripheral blood pressures with intermediate cardiovascular phenotypes. Hypertens. Dallas Tex.

[64] Smulyan H, Safar ME (1997). Systolic blood pressure revisited. J. Am. Coll. Cardiol..

[65] Strandberg TE, Pitkala K (2003). What is the most important component of blood pressure: systolic, diastolic or pulse pressure?. Curr. Opin. Nephrol. Hypertens..

[66] Tanaka H (2016). Hemodynamic correlates of blood pressure in older adults: The atherosclerosis risk in communities (ARIC) study. J. Clin. Hypertens. Greenwich Conn.

[67] Marvanova M (2016). Drug-induced cognitive impairment: Effect of cardiovascular agents. Ment. Health Clin..

[68] Schultz BG, Patten DK, Berlau DJ (2018). The role of statins in both cognitive impairment and protection against dementia: a tale of two mechanisms. Transl. Neurodegener..

[69] Yang W, Luo H, Ma Y, Si S, Zhao H (2021). Effects of antihypertensive drugs on cognitive function in elderly patients with hypertension: A review. Aging Dis..

[70] Rönnlund M, Nyberg L, Bäckman L, Nilsson L-G (2005). Stability, growth, and decline in adult life span development of declarative memory: Cross-sectional and longitudinal data from a population-based study. Psychol. Aging.

[71] Taylor JR (2017). The Cambridge Centre for Ageing and Neuroscience (Cam-CAN) data repository: Structural and functional MRI, MEG, and cognitive data from a cross-sectional adult lifespan sample. Neuroimage.

[72] Laborde S, Mosley E, Thayer JF (2017). Heart rate variability and cardiac vagal tone in psychophysiological research—recommendations for experiment planning, data analysis, and data reporting. Front. Psychol..

[73] Zulfiqar U, Jurivich DA, Gao W, Singer DH (2010). Relation of high heart rate variability to healthy longevity. Am. J. Cardiol..

[74] Goldberger AL (2000). PhysioBank, PhysioToolkit, and PhysioNet. Circulation.

[75] Vest AN (2018). An open source benchmarked toolbox for cardiovascular waveform and interval analysis. Physiol. Meas..

[76] Baddeley A, Emslie H, Nimmo-Smith I (1993). The Spot-the-Word test: A robust estimate of verbal intelligence based on lexical decision. Br. J. Clin. Psychol..

[77] Hodges, J. R. *Neurological aspects of dementia and normal aging. Dementia Normal aging*, 118–129. (1994).

[78] Cattell, R. B. *Abilities: their structure, growth, and action*. xxii, 583 (Houghton Mifflin, 1971).

[79] Kievit RA (2014). Distinct aspects of frontal lobe structure mediate age-related differences in fluid intelligence and multitasking. Nat. Commun..

[80] R Core Team. A Language and Environment for Statistical Computing R Foundation for Statistical Computing, Vienna, Austria. (2020).

[81] Rosseel Y (2012). lavaan: An R package for structural equation modeling. J. Stat. Softw..

[82] Little TD, Cunningham WA, Shahar G, Widaman KF (2002). To parcel or not to parcel: Exploring the question, weighing the merits. Struct. Equ. Model..

[83] Little TD, Lindenberger U, Nesselroade JR (1999). On selecting indicators for multivariate measurement and modeling with latent variables: When ‘good’ indicators are bad and ‘bad’ indicators are good. Psychol. Methods.

[84] Watkins MW (2018). Exploratory factor analysis: A guide to best practice. J. Black Psychol..

[85] Revelle, W. psych: Procedures for Personality and Psychological Research, Northwestern University, Evanston, Illinois, USA, https://CRAN.R-project.org/package=psych. (2017).

[86] Cattell RB (1943). The measurement of adult intelligence. Psychol. Bull..

[87] Cook RD (1977). Detection of influential observation in linear regression. Technometrics.

[88] Venables W. N. & Ripley, B. D. *Modern Applied Statistics with S*. 4th edition. (Springer, 2002).

[89] Klassen SA, Joyner MJ, Baker SE (2021). The impact of ageing and sex on sympathetic neurocirculatory regulation. Semin. Cell Dev. Biol..

[90] Reas ET (2021). Age and sex differences in the associations of pulse pressure with white matter and subcortical microstructure. Hypertension.

[91] Thayer JF, Mather M, Koenig J (2021). Stress and aging: A neurovisceral integration perspective. Psychophysiology.

[92] Chen W (2000). Age-related patterns of the clustering of cardiovascular risk variables of syndrome X from childhood to young adulthood in a population made up of black and white subjects: The Bogalusa Heart Study. Diabetes.

[93] Goodman E, Dolan LM, Morrison JA, Daniels SR (2005). Factor analysis of clustered cardiovascular risks in adolescence. Circulation.

[94] Khader YS (2011). Factor analysis of cardiometabolic risk factors clustering in children and adolescents. Metab. Syndr. Relat. Disord..

[95] Mayer-Davis EJ (2009). Cardiovascular disease risk factors in youth with type 1 and type 2 diabetes: Implications of a factor analysis of clustering. Metab. Syndr. Relat. Disord..

[96] Wills AK (2011). Life course trajectories of systolic blood pressure using longitudinal data from eight UK cohorts. PLoS Med..

[97] Levin RA, Carnegie MH, Celermajer DS (2020). Pulse pressure: An emerging therapeutic target for dementia. Front. Neurosci..

[98] Wåhlin A, Nyberg L (2019). At the heart of cognitive functioning in aging. Trends Cogn. Sci..

[99] Malik M (1996). Heart rate variability: Standards of measurement, physiological interpretation, and clinical use. Eur. Heart J..

[100] Porges SW (2003). The polyvagal theory: Phylogenetic contributions to social behavior. Physiol. Behav..

[101] Schroeder EB (2003). Hypertension, blood pressure, and heart rate variability. Hypertension.

[102] Laborde S, Mosley E, Mertgen A (2018). Vagal tank theory: The three rs of cardiac vagal control functioning – resting, reactivity, and recovery. Front. Neurosci..

[103] Liu KY, Elliott T, Knowles M, Howard R (2022). Heart rate variability in relation to cognition and behavior in neurodegenerative diseases: A systematic review and meta-analysis. Ageing Res. Rev..

[104] Kimhy D (2013). The association of cardiac vagal control and executive functioning—Findings from the MIDUS study. J. Psychiatr. Res..

[105] Mann SL, Selby EA, Bates ME, Contrada RJ (2015). Integrating affective and cognitive correlates of heart rate variability: A structural equation modeling approach. Int. J. Psychophysiol..

[106] Stenfors CUD, Hanson LM, Theorell T, Osika WS (2016). Executive cognitive functioning and cardiovascular autonomic regulation in a population-based sample of working adults. Front. Psychol..

[107] Holzman JB, Bridgett DJ (2017). Heart rate variability indices as bio-markers of top-down self-regulatory mechanisms: A meta-analytic review. Neurosci. Biobehav. Rev..

[108] Mitchell GF (2008). Effects of central arterial aging on the structure and function of the peripheral vasculature: Implications for end-organ damage. J. Appl. Physiol..

[109] Obisesan TO (2008). High blood pressure, hypertension, and high pulse pressure are associated with poorer cognitive function in persons aged 60 and older: The third national health and nutrition examination survey. J. Am. Geriatr. Soc..

[110] Webb AJS (2012). Increased cerebral arterial pulsatility in patients with leukoaraiosis. Stroke.

[111] Bethlehem RAI (2020). Dispersion of functional gradients across the adult lifespan. Neuroimage.

[112] Tibon R (2021). Transient neural network dynamics in cognitive ageing. Neurobiol. Aging.

[113] Tsvetanov KA (2016). Extrinsic and intrinsic brain network connectivity maintains cognition across the lifespan despite accelerated decay of regional brain activation. J. Neurosci..

[114] Tsvetanov KA (2018). Activity and Connectivity differences underlying inhibitory control across the adult life span. J. Neurosci..

[115] Tsvetanov KA (2021). Brain functional network integrity sustains cognitive function despite atrophy in presymptomatic genetic frontotemporal dementia. Alzheimers Dement..

[116] Hughes TM (2013). Pulse wave velocity is associated with β-amyloid deposition in the brains of very elderly adults. Neurology.

[117] Rodrigue KM (2013). Risk factors for β-amyloid deposition in healthy aging: Vascular and genetic effects. JAMA Neurol..

[118] Stone J, Johnstone DM, Mitrofanis J, O’Rourke M (2015). The mechanical cause of age-related dementia (Alzheimer’s disease): The brain is destroyed by the pulse. J. Alzheimers Dis. JAD.

[119] Nation DA (2015). Pulse pressure in relation to tau-mediated neurodegeneration, cerebral amyloidosis, and progression to dementia in very old adults. JAMA Neurol..

[120] de Montgolfier O (2019). High systolic blood pressure induces cerebral microvascular endothelial dysfunction, neurovascular unit damage, and cognitive decline in mice. Hypertens. Dallas Tex.

[121] Wu, S. *et al. Cerebral blood flow predicts multiple demand network activity and fluid intelligence across the lifespan* (2021). 10.1101/2021.11.10.468042. 10.1016/j.neurobiolaging.2022.09.006 PMC7613814 36306687

[122] Langbaum JBS (2012). Blood pressure is associated with higher brain amyloid burden and lower glucose metabolism in healthy late middle-age persons. Neurobiol. Aging.

[123] Walker KA (2019). Association of midlife to late-life blood pressure patterns with incident dementia. JAMA.

[124] Simin M (2020). Cumulative blood pressure exposure during young adulthood and mobility and cognitive function in midlife. Circulation.

[125] McDonough IM, Cody SL, Harrell ER, Garrett SL, Popp TE (2022). Cognitive differences across ethnoracial category, socioeconomic status across the Alzheimer’s disease spectrum: Can an ability discrepancy score level the playing field?. Mem. Cognit..

[126] Köbe T (2021). Vascular risk factors are associated with a decline in resting-state functional connectivity in cognitively unimpaired individuals at risk for Alzheimer’s disease: Vascular risk factors and functional connectivity changes. Neuroimage.

[127] Stuhec M, Keuschler J, Serra-Mestres J, Isetta M (2017). Effects of different antihypertensive medication groups on cognitive function in older patients: A systematic review. Eur. Psychiatry J. Assoc. Eur. Psychiatr..

