# Supplementary material for: Distinct components of cardiovascular health are linked with age-related differences in cognitive abilities
Source: Sci Rep. 2023 Jan 18;13:978. doi: 10.1038/s41598-022-27252-1 (PMC9849401; doi:10.1038/s41598-022-27252-1)
Supplement: Supplementary file 1 — Supplementary Information. [file 41598_2022_27252_MOESM1_ESM.docx]

SUPPLEMENTARY MATERIAL


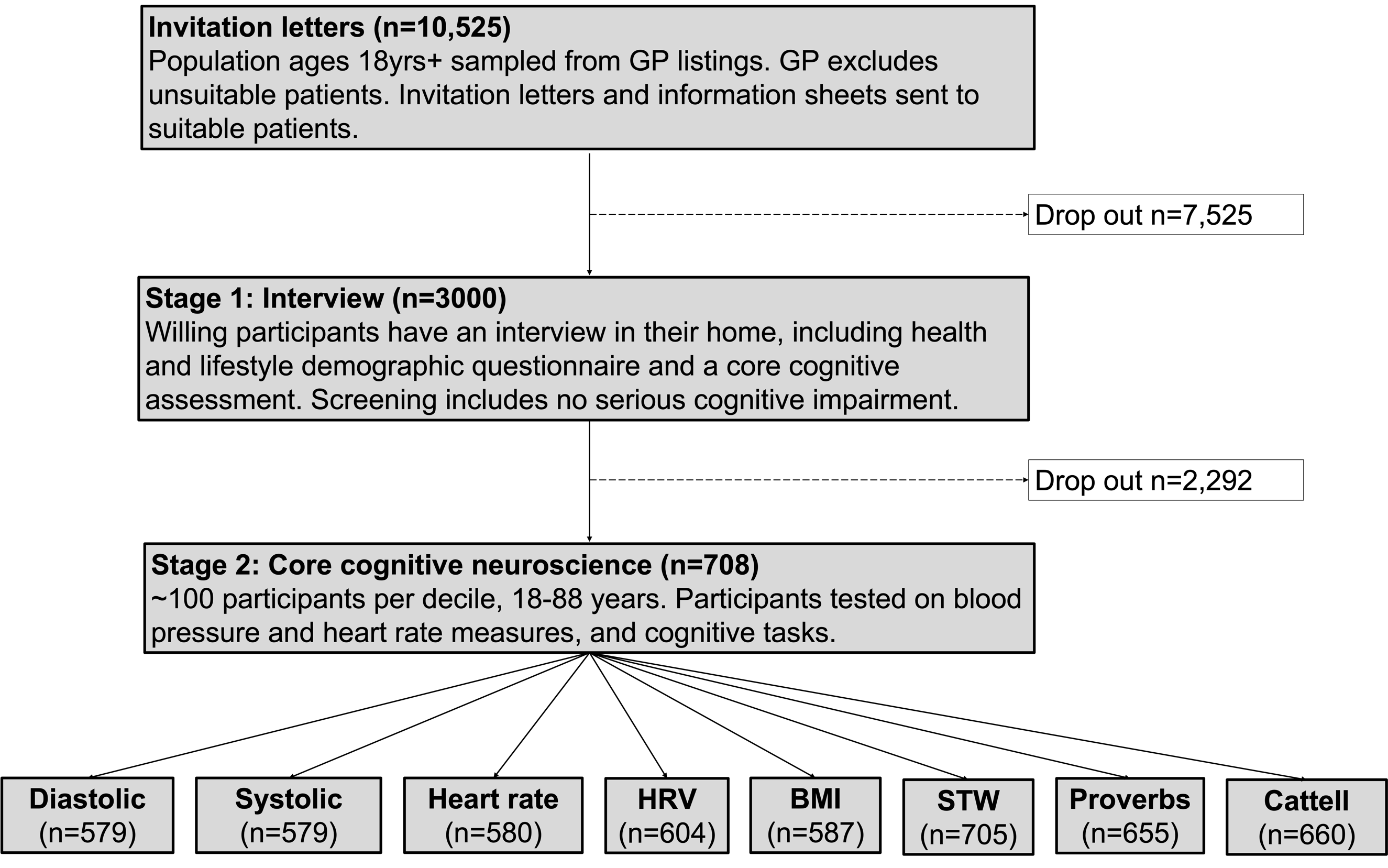


**Supplementary Figure 1**. Flowchart of participant recruitment in the Cam-CAN cohort, adapted from Shafto et al.^1^.

Abbreviations: HRV, heart rate variability, recorded at low and high frequencies; STW, spot the word.

**Supplementary Table 1. Cam-CAN exclusion criteria. Reproduced from Shafto et al.**^1^**.**

| Exclusion category | Exclusion criteria |
| --- | --- |
| Cognitive health | MMSE score 24 or less (calculated in interview)  Missing MMSE scores (assumed to be 24 or less)  Severe memory defect Consent difficulties for next stage |
| Communication difficulties | Hearing problems (difficulty completing interview with hearing aid; inability to hear 35 db at 1000 Hz in either ear in interview functional hearing screening; hearing aid that cannot be removed)  Insufficient English language (native language not English and not bilingual English from birth)  Vision difficulties (correct near vision of 20/100 or worse with both eyes) |
| Medical problems (self-report of diagnosis) | Dementia diagnosis/Alzheimer’s Disease  Parkinson’s disease  Motor Neurone disease  Multiple Sclerosis  Cancer (history of brain tumour or chemotherapy/radiotherapy for any cancer in last 6 months)  Stroke  Encephalitis  Meningitis  Epilepsy  Head injury with serious results (coma, unconscious for >2 hrs, or skull fracture)  Recently diagnosed or uncontrolled high blood pressure  Pregnancy or trying to become pregnant  Current serious psychiatric conditions (bipolar disorder, schizophrenia, or psychosis) |
| Mobility problems | Restricted mobility which would prevent further participation  Inability to walk 10 metres |
| Substance abuse | Past or current treatment for drug abuse  Current drug usage  Refusal to answer substance abuse questions |
| Specific MRI/MEG safety and comfort exclusions | Heart operation  Blood vessel procedure or device (carotid artery vascular clamp; venous umbrella; stent, filter or coil; Swan-Ganz catheter; vascular access ports or catheters)  Neurostimulator or spinal fusion stimulator  Electrodes on body, head or brain  Pump, Implant or pacemaker  Brain Operation  Metal splinters in eye, head or ear  Shrapnel, buckshot or bullet in body  Wire sutures or surgical staples  Artificial joints that are MRI incompatible (jaw/maxillary reconstruction; shoulder prosthesis; any other joint replacement surgery in the last 3 months)  Bone fixation rods or plates in jaw, head, shoulders or spine  Non-removable dental brace  Non-removable prosthesis or removable eye prosthesis  Inability to lie flat for an hour  Claustrophobia  Body piercings that cannot be removed  IUD that is MRI incompatible  Transdermal delivery patches that cannot be removed  Tattoos on head face or neck |

Abbreviations: MMSE, Mini-Mental State Examination.

SUPPLEMENTARY SECTION A

Introduction

The winning 3-factor EFA model (Figure 2) was saturated and therefore lacked absolute fit indices. The robustness of this model was investigated using Exploratory Structural Equation Modelling (ESEM) ^2^. ESEM integrates confirmatory factor analysis and structural equation modelling to provide confirmatory tests of a priori factor structures. It allowed modelling of vascular and cognitive factors simultaneously. We hypothesised a winning model with three latent vascular factors ^3–7^, and with two latent cognitive factors representing the distinct domains of fluid and crystallized intelligence ^8–10^. We further hypothesised that the three latent vascular factors in the ESEM model would structurally resemble and correlate highly with the corresponding latent vascular factors produced in the main EFA analysis (Figure 2). This would evidence that a 3-factor structure robustly and reliably captures the vascular variables (blood pressure, pulse pressure, BMI, heart rate and heart rate variability). This could allow advances in the design of studies to understand the links between vascular health and cognitive ageing.

Methods

Participants with <2 observations in either vascular or cognitive variables were excluded (n=655). Confirmatory Factor Analysis produced latent factors, as in the main analysis (Figure 1). Then all vascular and cognitive variables were input to ESEM models, estimated with the Psych package ^11^. The ESEM models produced latent vascular factors and LCFs using only participants with complete data across all variables for each domain (latent vascular factors n=516, latent cognitive factors n=636). Iterative combinations of 1-4 latent vascular factors and 1-4 LCFs were explored. Model fit was evaluated according to the Bayesian Information Criterion (BIC), adjusted for sample size. From the winning model, subject scores were extracted that represent how each participant’s observed data loads onto the latent vascular factors. The latent vascular factors produced in the ESEM model were correlated with those produced in EFA.

Results

The winning ESEM model has 3 vascular and 2 cognitive factors, indicated as “3_2” in Supplementary Figure 2. The latent vascular factors produced in ESEM correlated highly those produced in EFA (r>0.99, p<0.001, in all instances; Supplementary Figure 3).

Discussion

The vascular structure is robustly and reliably produced in EFA and ESEM data-driven models. This provides strong support for the initial EFA model, in the absence of absolute fit indices. The EFA vascular structure could be further confirmed in future studies by expanding the model with additional variables, such as heart rate variability reactivity, orthostatic intolerance or pulse wave velocity. It would also be possible to extend the ESEM analysis by calculating the ability discrepancy score from the latent cognitive factors, which resemble crystallized and fluid intelligence ^10^. The discrepancy could then be related to latent vascular factors in multiple linear regression, as in the main analysis of the present study. However, this was not implemented because to model the vascular and cognitive factors simultaneously in ESEM, and to then relate them again in regression, is circular. In summary, the multifactorial vascular structure is robust and will inform the design of future studies on vascular and cognitive ageing.


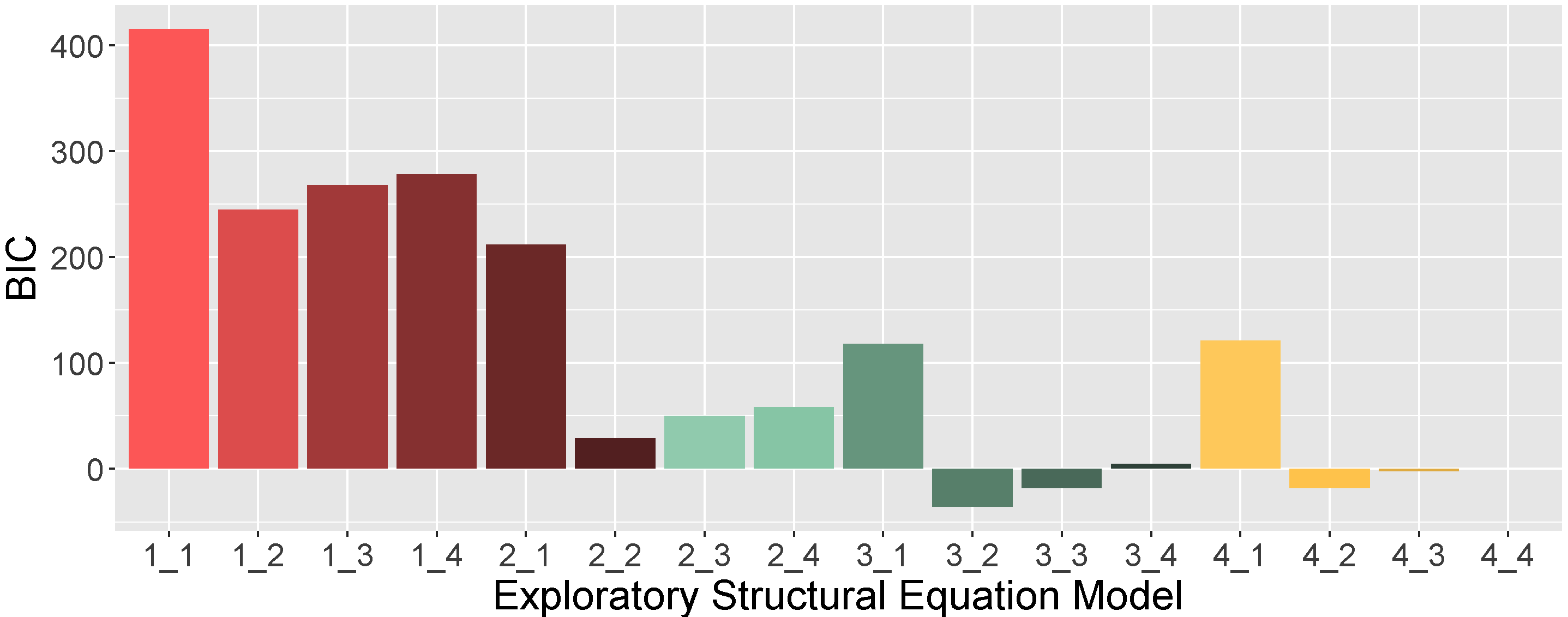


**Supplementary Figure 2.**A comparison of model fit for Exploratory Structural Equation Models. Individual models are each coloured and labelled on the x-axis with two digits, separated by an underscore, of which the first represents the number of vascular and the second the number of cognitive latent variables within a given model. Fit is measured by the Bayesian Information Criterion (BIC). Model “3_2” has the best overall fit and was selected for further examination.


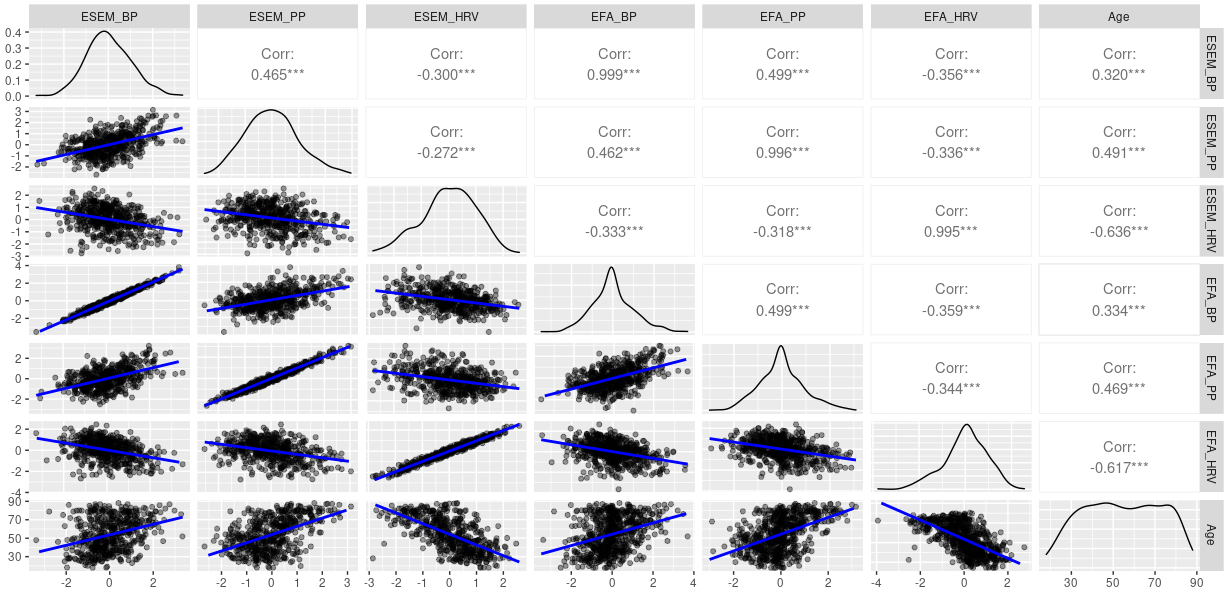


**Supplementary Figure 3**. Scatter plots (lower left), distributions (leading diagonal) and Pearson correlations (upper right) for latent vascular factors produced in ESEM and EFA, and age. Latent vascular factors are named according to the observed variable(s) predominantly expressed: blood pressure (BP), pulse pressure (PP) and heart rate variability (HRV). Scatter plots show linear associations (blue) and data intensity (greyscale). Stars indicate increasing significance on the correlations: ***, p<0.001; **, p<0.01; *, p<0.05.

Abbreviations: Corr, correlation coefficient; EFA, Exploratory Factor Analysis; ESEM, Exploratory Structural Equation Modelling.

SUPPLEMENTARY SECTION B

Introduction

The EFA vascular model was run across a broad age range (n=668, 18-88 years). It is possible that across the lifespan there are changes in elements of vascular health and the covariances between them. The mean age trends within these vascular observations could therefore influence the structure of the EFA model. To investigate the model’s robustness with age, we repeated the EFA model on groups of young (n=158, 18-37years), middle (n=311, 38-67 years) and old (n=199, 68-88 years) participants. We firstly evaluated whether EFA run across age specific sub-groups produced a similar model structure and composition, to when run across all participants simultaneously. We secondly investigated whether the multigroup EFA would replicate the regression results, showing relationships between vascular factors and the ability discrepancy score. Overall, this gave a robust investigation into the effects of age on our model of vascular health in this cross-sectional and lifespan adult cohort.

Methods

EFA was run in the Lavaan package^12^. In the initial EFA run across the whole sample, vascular observations were first standardized (mean=0, standard deviation=1) across the whole sample. For the multigroup EFA run over age specific sub-groups, vascular observations were standardized across sub-groups. Mean centring within sub-groups removed mean age trends in the vascular variables, which were important for the subsequent linear regression models. Therefore, the latent vascular factors produced in the multigroup EFA had age-group means re-injected into them, before regression. This was done by calculating the mean of a given latent vascular factor, as produced in the whole sample EFA, using only participants corresponding to a given age group from the multigroup EFA. The resulting mean was added to the factor scores for that age group. The latent vascular factors for a given age group were then stacked, correlated with latent factors produced in the whole sample EFA, and input to regression models (see Supplementary Section D). Inputting the multigroup EFA factors into the regression models allowed us to investigate whether the relationship between vascular factors and cognitive ability discrepancy remained consistent when the vascular factors were modelled in age specific sub-groups.

Results
In the EFA models run across multiple age groups, the three-factor models fit best (Supplementary Table 2). The latent vascular factors produced in the age group specific and whole sample EFA models correlated highly (r>0.70, p<0.001) (Supplementary Figure 4). The highest correlation was for latent vascular factor 2 (r=0.97, p<0.001). Factor scores from the multigroup EFA were extracted and input to the regression models. Model comparisons showed that Model 2 fit the data best (Supplementary Table 3). In Model 2, the latent vascular factor 2 significantly interacted with quadratic age to predict the ability discrepancy score (std β=0.082, SE=0.032, p=0.011) (Supplementary Table 4).

Discussion

The three-factor vascular structure was consistently produced in EFA models across both the whole sample and age sub-groups, and in the ESEM approach (Supplementary Section A). This gives strong support for multiple vascular signals. The composition of the three latent vascular factors was robust between EFA models on the whole sample and on sub-groups. This suggests that factor composition was not substantially influenced by age. Finally, the relationship of these latent vascular factors to the ability discrepancy score in regression models was consistent across the whole sample and age sub-group EFA models. In both approaches, the latent vascular factor 2, which predominantly expressed pulse pressure, interacted with quadratic age to significantly predict the ability discrepancy. In summary, the vascular model was robust to age effects and there was a significant effect of pulse pressure on cognitive change throughout the lifespan.

**Supplementary Table 2.** Model comparisons for the EFA run on young (n=158, 18-37years), middle (n=311, 38-67 years) and old (n=199, 68-88 years) participants. Note the limitation that there was poor convergence in all two-factor models, and in the three-factor models for young and middle age groups.

|  | Young | Middle | Old |
| --- | --- | --- | --- |
| Model 1 vs 2 | ∆ *X^2^* = 37.12, p<0.001 | ∆ *X^2^* = 90.65, p<0.001 | ∆ *X^2^* = 109.97, p<0.001 |
| Model 2 vs 3 | ∆ *X^2^* = 14.26, p=0.01 | ∆ *X^2^* = 70.51, p<0.001 | ∆ *X^2^* = 30.03, p<0.001 |

**
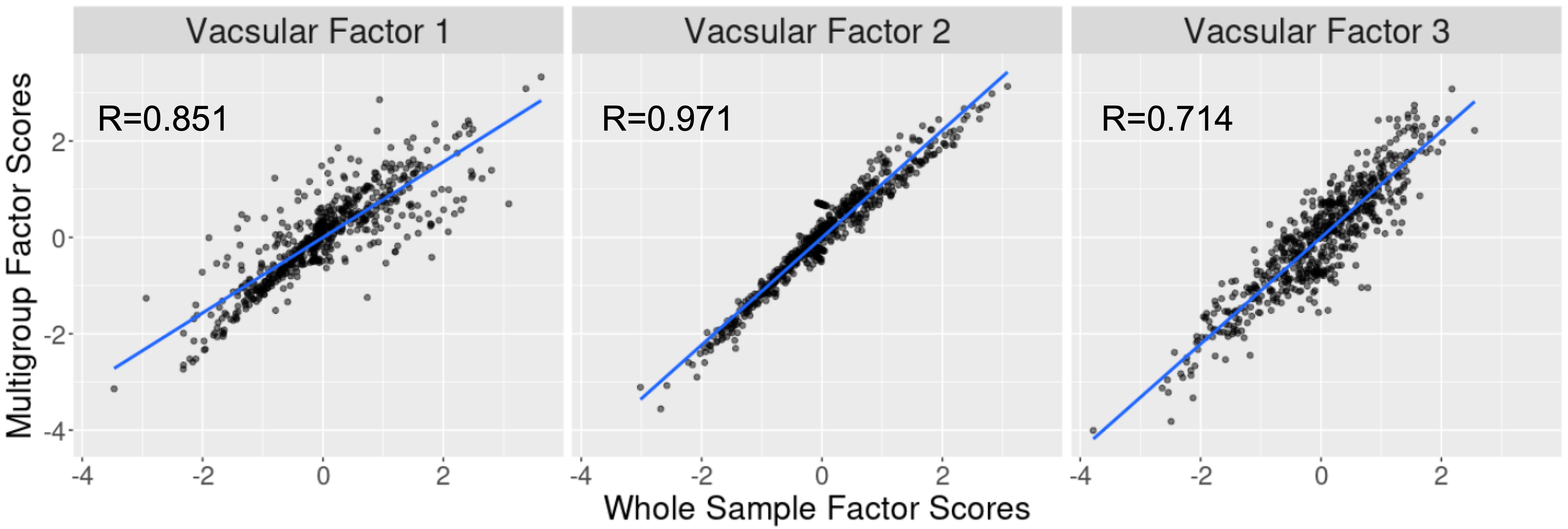
**

**Supplementary Figure 4.** Correlations of latent vascular factors 1-3, produced in the whole sample and multi-group EFA approaches. Scatter plots show linear associations (blue) and data intensity (greyscale). All correlations achieved significance of p<0.001.

**Supplementary Table 3.** Results of model comparisons, using AIC and BIC, and the sum of squares derived from ANOVA comparisons. Here, regression models were run using latent vascular factors produced in EFA run over three age specific sub-groups.

|  | Difference in AIC | Difference in BIC | Difference in Sum  Of Squares |
| --- | --- | --- | --- |
| Model 1 vs 2 | 305.79 | 269.91 | 207.95 |
| Model 2 vs 3 | -17.49 | -214.81 | 33.46 |
| Model 2 vs 4 | -7.79 | -39.18 | 3.09 |
| Model 2 vs 5 | -14.95 | -50.83 | 0.52 |

Abbreviations: AIC, Akaike information criterion; BIC, Bayesian information criterion.

**Supplementary Table 4.** Results of Model 2 (n=655, DoF=634, residual standard error =0.65). Significant effects (p<0.05) are shown in bold. Here, regression models were run using latent vascular factors produced in EFA run over three age specific sub-groups.

| Predictors | Standard β | Standard Error | Confidence Intervals | p |
| --- | --- | --- | --- | --- |
| LVF1 | -0.03 | 0.03 | -0.09 – 0.04 | 0.44 |
| LVF2 | -0.02 | 0.03 | -0.08 – 0.05 | 0.56 |
| LVF3 | 0.01 | 0.03 | -0.08 – 0.06 | 0.75 |
| Age | 0.71 | 0.04 | 0.62 – 0.79 | **<0.001** |
| Age^2^ | 0.03 | 0.04 | -0.04 – 0.10 | 0.34 |
| LVF1.Age | -0.04 | 0.03 | -0.10 – 0.03 | 0.29 |
| LVF1.Age^2^ | -0.04 | 0.03 | -0.10 – 0.02 | 0.19 |
| LVF2.Age | <0.01 | 0.03 | -0.07 – 0.06 | 0.98 |
| LVF2.Age^2^ | 0.08 | 0.03 | 0.02 – 0.15 | **0.01** |
| LVF3.Age | <0.01 | 0.03 | -0.06 – 0.07 | 0.94 |
| LVF3.Age^2^ | -0.01 | 0.03 | -0.07 – 0.05 | 0.78 |
| Sex | 0.01 | 0.06 | -0.11 – 0.12 | 0.88 |
| Education |  |  |  |  |
| No qualifications tried | -0.86 | 0.59 | -2.02 – 0.31 | 0.15 |
| GCSEs / O-levels | -0.96 | 0.59 | -2.11 – 0.19 | 0.10 |
| A-levels | -0.72 | 0.58 | -1.87 – 0.43 | 0.22 |
| Degree | -0.59 | 0.58 | -1.73 – 0.55 | 0.31 |
| General Health |  |  |  |  |
| No response given | 0.31 | 0.58 | -0.83 – 1.44 | 0.60 |
| Excellent | 0.66 | 0.58 | -0.48 – 1.80 | 0.26 |
| Good | 0.71 | 0.58 | -0.44 – 1.85 | 0.23 |
| Fair | 0.66 | 0.59 | -0.49 – 1.82 | 0.26 |
| Poor | 1.03 | 0.64 | -0.22 – 2.28 | 0.11 |

Abbreviations: LVF1-3, latent vascular factors.

SUPPLEMENTARY SECTION C

Introduction

The discrepancy between an individual’s score on fluid versus crystallized intelligence was used to approximate cognitive decline, on the basis of three assumptions. Firstly, it assumed that the measurement of fluid and crystallized intelligence was invariant to age. We modelled fluid and crystallized intelligence in confirmatory factor analysis, with two latent variables (Figure 2). This model was run across a broad age range (n=668, 18-88 years). It is possible that across the lifespan there are changes in elements of cognition which effect the measurement of fluid and crystallized intelligence, and therefore the composition of factors in our model. To investigate the model’s robustness to age, we ran Moderated Non-Linear Factor Analysis^13^. This method of invariance testing is well suited to examine invariance with age because it accommodates continuous variables, which is not possible in multigroup confirmatory factor analysis. The second assumption on the discrepancy between fluid and crystallized intelligence was that the two are highly correlated in youth. The third assumption was that crystallized measures do not change with age. The second and third assumptions were investigated with visualisations and correlations.

Methods

Measurement invariance with age on the two-factor model of crystallized and fluid intelligence was assessed using Moderated Non-Linear Factor Analysis, implemented in the OpenMx package in R^13^. Invariance was assessed over an unconstrained model where intercepts and factor loadings were free to vary, versus a constrained model where these effects were fixed. If the free model fit better than the constrained, when compared with the likelihood-ratio test (p<0.05), invariance was rejected.

Analysis scripts can be downloaded: <https://github.com/DebsKing/Distinct_Vascular_Components_Relate_To_Cognition>.

Results

Measurement invariance tested with moderated non-linear factor analysis gave evidence for invariance, which is to say that the two-factor model of fluid and crystallized intelligence

did not vary substantially with age (∆ *χ*^2^(8) =14.51, p=0.07). Additional fit metrics are not reported due to limited degrees of freedom and in line with the tutorial^13^. Addressing the second assumption, fluid and crystallized intelligence correlated highly in young adults (n=158, r=0.55, p<0.001). Although there was also a high correlation across the group of old adults (n=205, r=0.49, p<0.001), the variance in the distribution increased, and the individuals with lower correlations likely corresponded to those with a higher ability discrepancy. On the third assumption, crystallized intelligence remains stable with age (Supplementary Figure 5).


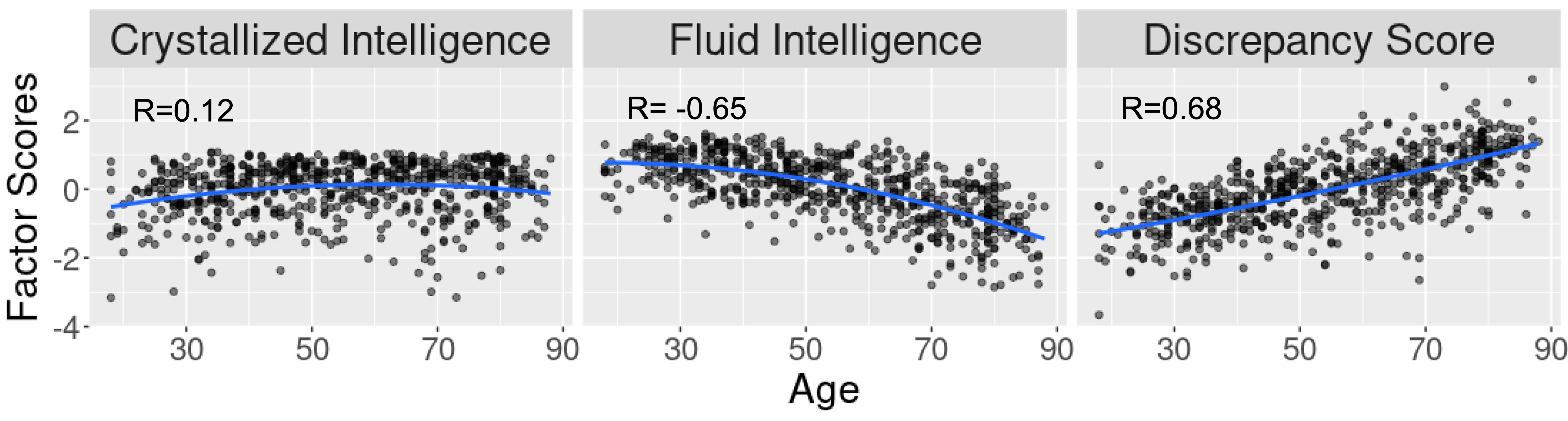


**Supplementary Figure 5.** Crystallized intelligence correlates poorly with age and remains relatively constant compared to fluid intelligence and the ability discrepancy (n=678). Scatter plots show associations (blue) and data intensity (greyscale).

Discussion

The three assumptions on the discrepancy between an individual’s score on fluid versus crystallized intelligence were met. The measurement model of fluid and crystallized intelligence was invariant to age; crystallized and fluid intelligence correlated highly in young adults; and crystallized intelligence remained stable with age.

SUPPLEMENTARY SECTION D

Five models were used to test different hypotheses. The models are as follows.
In the following model syntax, latent vascular factors are abbreviated to LVF1-3.

Model 1: y ~ β_0_ + β_1_.LVF1 + β_2_.LVF2 + β_3_.LVF3 + general health + covs + ɛ

where “y” is ability discrepancy and “covs” are the covariates of no interest, i.e, sex and education, “β” are the parameter estimates (coefficients) and “ɛ” is the residual error.

Model 2: y ~ β_0_ + β_1_.LVF1 + β_2_.LVF2 + β_3_.LVF3
 + β_4_.Age + β_5_.Age^2^
 + β_6_.LVF1.Age + β_7_.LVF2.Age + β_8_.LVF3.Age
 + β_9_.LVF1.Age^2^ + β_10_.LVF2.Age^2^ + β_11_.LVF3.Age^2^ + general health + covs + ɛ

Model 3: y ~ β_0_ + β_1_.LVF1 + β_2_.LVF2 + β_3_.LVF3
 + β_4_.Age + β_5_.Age^2^
 + β_6_.Anti-Hypertensives + β_7_.Beta blockers + β_8_.Diuretics
 + β_9_.Dyslipidemics
 + β_10_.LVF1.Age + β_11_.LVF2.Age + β_12_.LVF3.Age
 + β_13_.LVF1.Age^2^ + β_14_.LVF2.Age^2^ + β_15_.LVF3.Age^2^
 + β_16_.LVF1.Anti-Hypertensives + β_17_.LVF2.Anti-Hypertensives
 + β_18_.LVF3.Anti-Hypertensives
 + β_19_.LVF1.Beta blockers + β_20_.LVF2.Beta blockers
 + β_21_.LVF3.Beta blockers
 + β_22_.LVF1.Diuretics + β_23_.LVF2.Diuretics + β_24_.LVF3.Diuretics
 + β_25_.LVF1. Dyslipidemics + β_26_.LVF2. Dyslipidemics
 + β_27_.LVF3. Dyslipidemics

+ β_28_.LVF1.Anti-Hypertensives.Age + β_29_.LVF2.Anti-Hypertensives.Age
 + β_30_.LVF3.Anti-Hypertensives.Age
 + β_31_.LVF1.Beta blockers.Age + β_32_.LVF2.Beta blockers.Age
 + β_33_.LVF3.Beta blockers.Age
 + β_34_.LVF1.Diuretics.Age + β_35_.LVF2.Diuretics.Age
 + β_36_.LVF3.Diuretics.Age
 + β_37_.LVF1. Dyslipidemics.Age + β_38_.LVF2. Dyslipidemics.Age
 + β_39_.LVF3. Dyslipidemics.Age
 + β_40_.LVF1.Anti-Hypertensives.Age^2^ + β_41_.LVF2.Anti-Hypertensives.Age^2^
 + β_42_.LVF3.Anti-Hypertensives.Age^2^
 + β_43_.LVF1.Beta blockers.Age^2^ + β_44_.LVF2.Beta blockers.Age^2^
 + β_45_.LVF3.Beta blockers.Age^2^
 + β_46_.LVF1.Diuretics.Age^2^ + β_47_.LVF2.Diuretics.Age^2^
 + β_48_.LVF3.Diuretics.Age^2^
 + β_49_.LVF1. Dyslipidemics.Age^2^ + β_50_.LVF2. Dyslipidemics.Age^2^

+ β_51_.LVF3. Dyslipidemics.Age^2^

+ β_52_.Anti-Hypertensives.Age + β_53_.Beta blockers.Age
 + β_54_.Diuretics.Age + β_55_. Dyslipidemics.Age
 + β_56_.Anti-Hypertensives.Age^2^ + β_57_.Beta blockers.Age^2^
 + β_58_.Diuretics.Age^2^ + β_59_. Dyslipidemics.Age^2^ + covs + ɛ

Model 4: y ~ β_0_ + β_1_.LVF1 + β_2_.LVF2 + β_3_.LVF3
 + β_4_.Age + β_5_.Age^2^ + β_6_.Sex
 + β_7_.LVF1.Age + β_8_.LVF2.Age + β_9_.LVF3.Age
 + β_10_.LVF1.Age^2^ + β_11_.LVF2.Age^2^ + β_12_.LVF3.Age^2^ + β_13_.LVF1.Sex + β_14_.LVF2.Sex + β_15_.LVF3.Sex
 + β_16_.LVF1.Age.Sex + β_17_.LVF2.Age.Sex + β_18_.LVF3.Age.Sex
 + β_19_.LVF1.Age^2^.Sex + β_20_.LVF2.Age^2^.Sex + β_21_.LVF3.Age^2^.Sex
 + β_22_.Age.Sex + β_23_.Age^2^.Sex + covs + ɛ

Model 5: y ~ β_0_ + β_1_.LVF1 + β_2_.LVF2 + β_3_.LVF3
 + β_4_.Age + β_5_.Age^2^
 + β_6_.LVF1.Age + β_7_.LVF2.Age + β_8_.LVF3.Age
 + β_9_.LVF1.Age^2^ + β_10_.LVF2.Age^2^ + β_11_.LVF3.Age^2^
 + β_12_.LVF1.LVF2 + β_13_.LVF1.LVF3 + β_14_.LVF2.LVF3
 + β_15_.LVF1.LVF2.LVF3
 + β_16_.LVF1.LVF2.Age + β_17_.LVF1.LVF3.Age + β_18_.LVF2.LVF3.Age
 + β_19_.LVF1.LVF2.LVF3.Age
 + β_20_.LVF1.LVF2.Age^2^ + β_21_.LVF1.LVF3.Age^2^ + β_22_.LVF2.LVF3.Age^2^ + β_23_.LVF1.LVF2.LVF3.Age^2^ + covs + ɛ


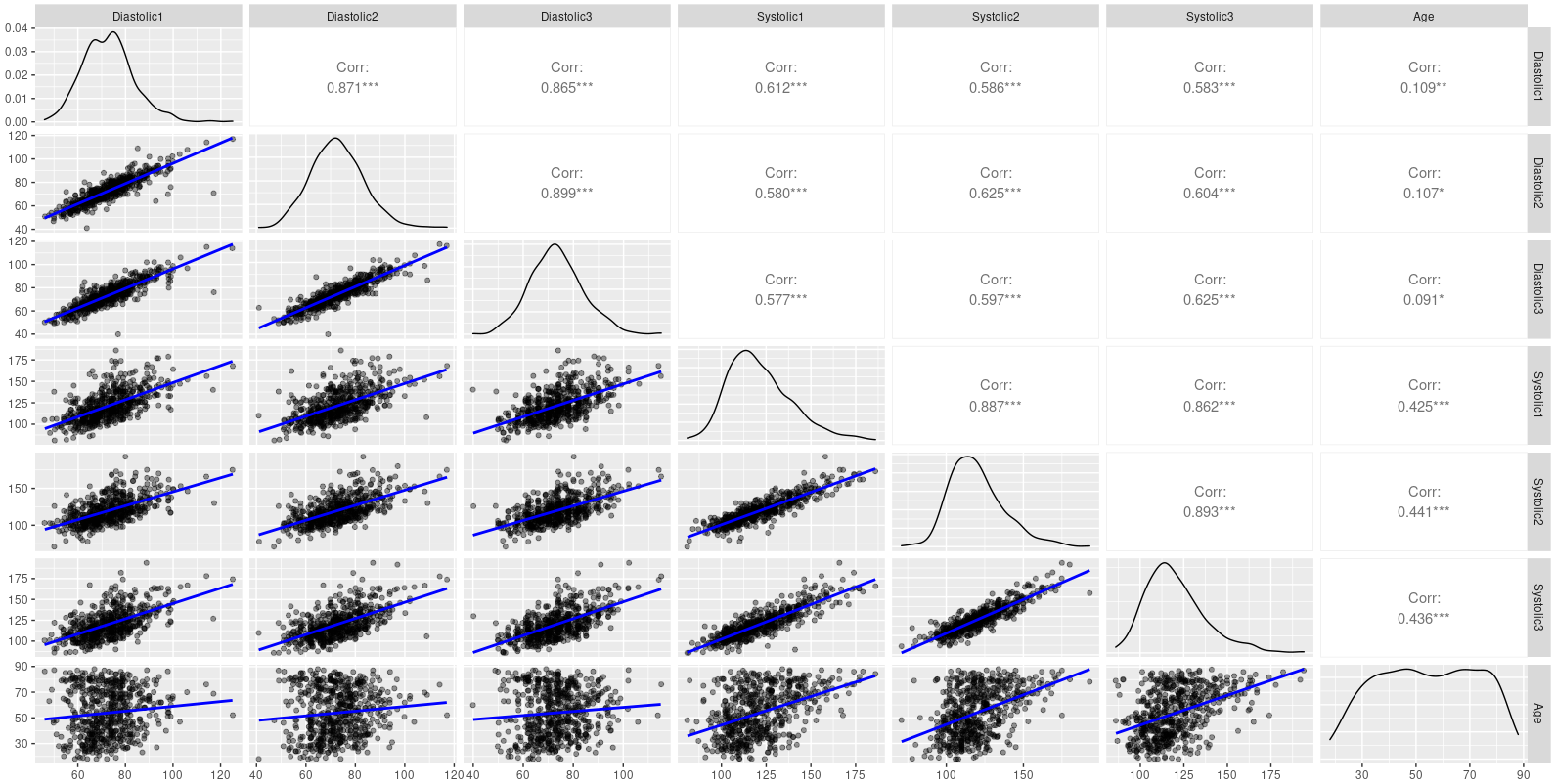


**Supplementary Figure 6**. Scatter plots (lower left), distributions (leading diagonal) and Pearson correlations (upper right) for age and diastolic and systolic blood pressure, across the first (n=578), second (n=579) and third (n=577) blood pressure recordings. Hypertension is clinically diagnosed when diastolic >90 mmHg and systolic >140 mmHg, or systolic >150mmHg for individuals aged over 80 ^14^. Scatter plots show linear associations (blue) and data intensity (greyscale). Stars indicate increasing significance on the correlations: ***, p<0.001; **, p<0.01; *, p<0.05.

Abbreviation: Corr, correlation coefficient.


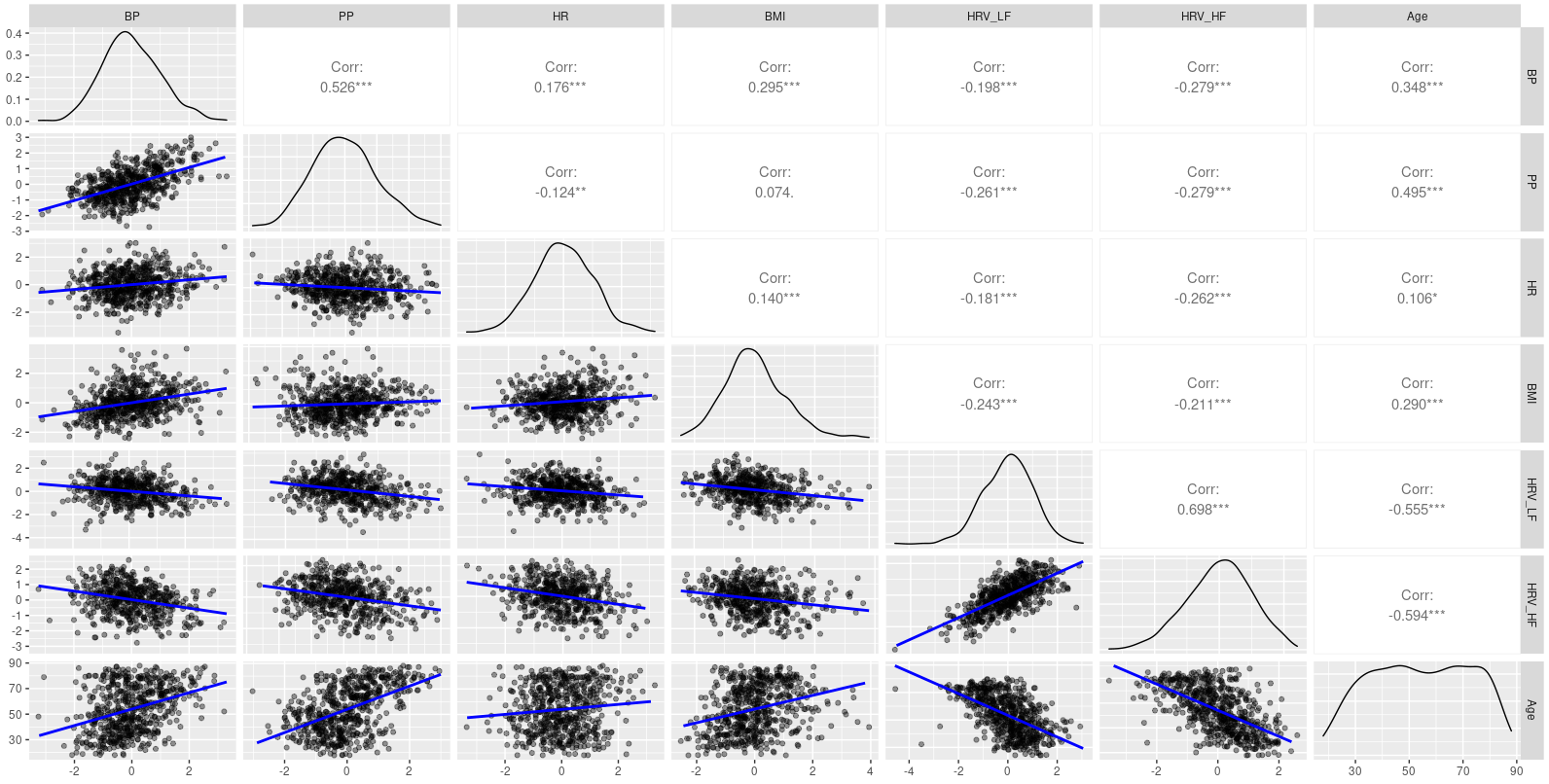


**Supplementary Figure 7**. Scatter plots (lower left), distributions (leading diagonal) and Pearson correlations (upper right) for vascular factors and age. Scatter plots show linear associations (blue) and data intensity (greyscale). Stars indicate increasing significance on the correlations: ***, p<0.001; **, p<0.01; *, p<0.05.

Abbreviations: BMI, body mass index; BP, total blood pressure; Corr, correlation coefficient; HR, heart rate; HRV_HF, heart rate variability at high frequency; HRV_LF, heart rate variability at low frequency; PP, pulse pressure.


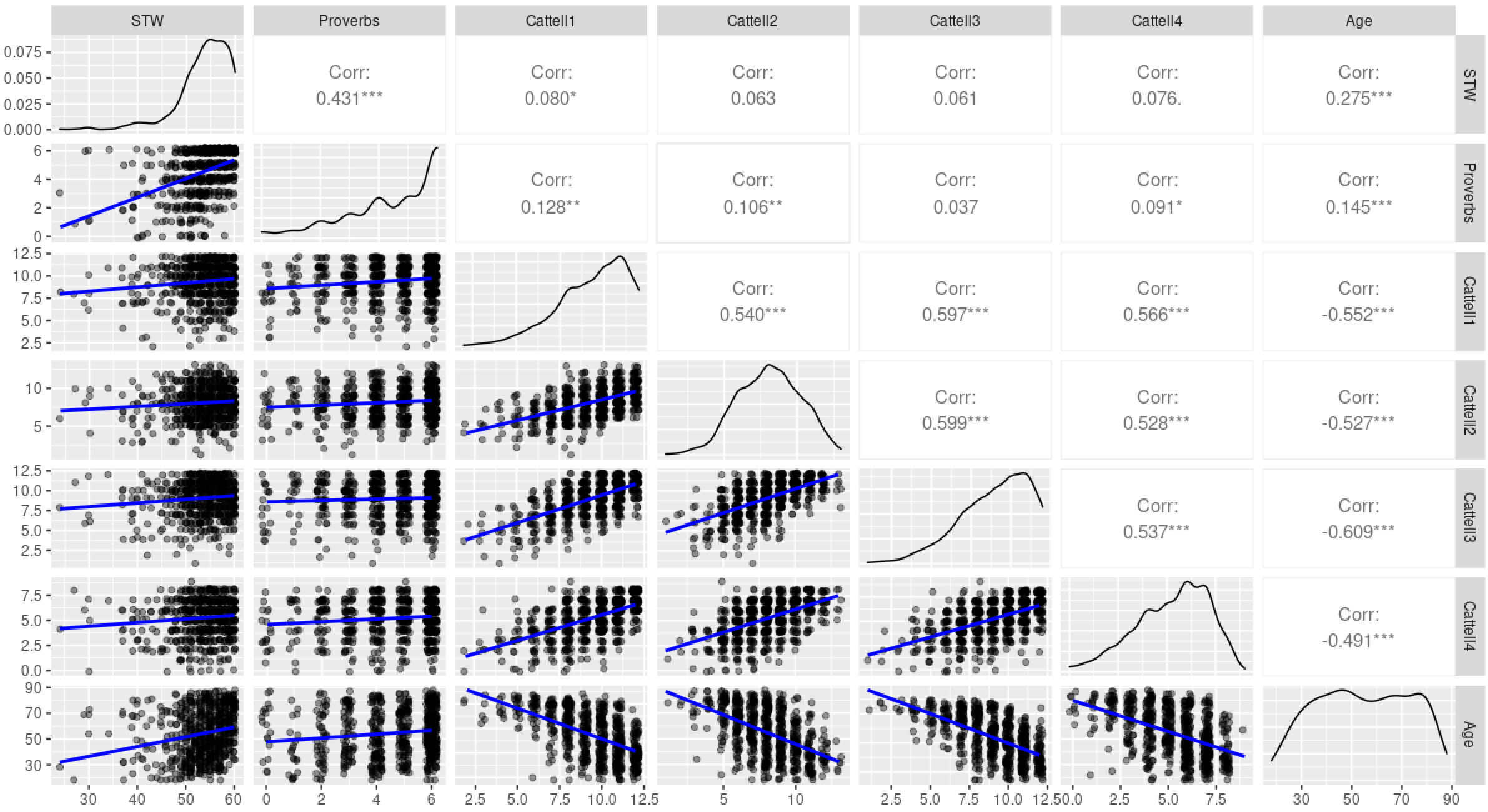


**Supplementary Figure 8**. Scatter plots (lower left), distributions (leading diagonal) and Spearman correlations (upper right) for cognitive observed variables and age. Scatter plots show linear associations (blue) and data intensity (greyscale). Stars indicate increasing significance on the correlations: ***, p<0.001; **, p<0.01; *, p<0.05.

Abbreviations: Cattell 1-4, sub-scores across the four Cattell tasks; Corr, correlation coefficient; STW, spot the word.

**Supplementary Table 5.** Loadings of observed variables onto the latent variables in the winning 3 factor EFA model of vascular health (n=668).

| Predictors | LVF1 | LVF2 | LVF3 |
| --- | --- | --- | --- |
| HRV HF | -0.03 | < -0.01 | 0.85 |
| HRV LF | 0.06 | -0.05 | 0.82 |
| BP | 0.98 | 0.04 | 0.02 |
| BMI | 0.30 | -0.15 | -0.22 |
| PP | 0.06 | 0.91 | -0.05 |
| HR | 0.28 | -0.37 | -0.29 |

Abbreviations: BMI, body mass index; BP, total blood pressure; HR, heart rate; HRV HF, heart rate variability at high frequency; HRV LF, heart rate variability at low frequency; LVF, latent vascular factor; PP, pulse pressure.

**Supplementary Table 6.** Results of Model 1 (n=655, DoF=642, residual standard error =0.82). Significant effects (p<0.05) are shown in bold.

| Predictors | Standard β | Standard Error | Confidence Intervals | p |
| --- | --- | --- | --- | --- |
| LVF1 | <0.01 | 0.04 | -0.08 – 0.08 | 0.87 |
| LVF2 | 0.19 | 0.04 | 0.11 – 0.28 | **<0.001** |
| LVF3 | -0.35 | 0.04 | -0.42 – -0.27 | **<0.001** |
| Sex | 0.06 | 0.07 | -0.09 – 0.20 | 0.45 |
| Education |  |  |  |  |
| No qualifications tried | -0.20 | 0.75 | -1.69 – 1.28 | 0.79 |
| GCSEs / O-levels | -0.70 | 0.75 | -2.17 – 0.77 | 0.35 |
| A-levels | -0.42 | 0.74 | -1.88 – 1.05 | 0.58 |
| Degree | -0.42 | 0.74 | -1.88 – 1.03 | 0.57 |
| General Health |  |  |  |  |
| No response given | 0.15 | 0.74 | -1.30 – 1.60 | 0.84 |
| Excellent | 0.51 | 0.74 | -1.01 – 1.96 | 0.55 |
| Good | 0.45 | 0.74 | -1.01 – 1.91 | 0.77 |
| Fair | 0.22 | 0.75 | -1.26 – 1.69 | 0.77 |
| Poor | 0.66 | 0.81 | -0.93 – 2.25 | 0.42 |

Abbreviations: LVF1-3, latent vascular factors.

**Supplementary Table 7.** Results of Model 2 (n=655, DoF=634, residual standard error =0.65). Significant effects (p<0.05) are shown in bold.

| Predictors | Standard β | Standard Error | Confidence Intervals | p |
| --- | --- | --- | --- | --- |
| LVF1 | <0.01 | 0.04 | -0.06 – 0.08 | 0.82 |
| LVF2 | -0.02 | 0.04 | -0.10 – 0.05 | 0.59 |
| LVF3 | 0.02 | 0.04 | -0.06 – 0.09 | 0.62 |
| Age | 0.71 | 0.05 | 0.62 – 0.80 | **<0.001** |
| Age^2^ | 0.04 | 0.04 | -0.04 – 0.11 | 0.35 |
| LVF1.Age | <0.01 | 0.03 | -0.07 – 0.07 | 0.93 |
| LVF1.Age^2^ | -0.03 | 0.03 | -0.09 – 0.04 | 0.43 |
| LVF2.Age | 0.01 | 0.04 | -0.07 – 0.08 | 0.89 |
| LVF2.Age^2^ | 0.08 | 0.04 | 0.01 – 0.15 | **0.03** |
| LVF3.Age | 0.01 | 0.04 | -0.06 – 0.09 | 0.73 |
| LVF3.Age^2^ | <0.01 | 0.04 | -0.07 – 0.07 | 0.99 |
| Sex | <0.01 | 0.06 | -0.11 – 0.12 | 0.94 |
| Education |  |  |  |  |
| No qualifications tried | -0.85 | 0.60 | -2.03 – 0.33 | 0.16 |
| GCSEs / O-levels | -0.95 | 0.59 | -2.11 – 0.22 | 0.11 |
| A-levels | -0.70 | 0.59 | -1.87 – 0.46 | 0.24 |
| Degree | -0.57 | 0.59 | -1.73 – 0.59 | 0.33 |
| General Health |  |  |  |  |
| No response given | 0.31 | 0.59 | -0.85 – 1.46 | 0.60 |
| Excellent | 0.63 | 0.59 | -0.53 – 1.79 | 0.29 |
| Good | 0.68 | 0.59 | -0.48 – 1.84 | 0.25 |
| Fair | 0.64 | 0.60 | -0.53 – 1.81 | 0.29 |
| Poor | 1.02 | 0.65 | -0.25 – 2.29 | 0.12 |

Abbreviations: LVF1-3, latent vascular factors.

**Supplementary Table 8**. Results of model comparisons, using AIC and BIC, and the sum of squares derived from ANOVA comparisons.

|  | Difference in AIC | Difference in BIC | Difference in Sum  Of Squares |
| --- | --- | --- | --- |
| Model 1 vs 2 | 279.66 | 243.79 | 187.92 |
| Model 2 vs 3 | -18.61 | -215.93 | 33.11 |
| Model 2 vs 4 | -9.47 | -40.87 | 2.27 |
| Model 2 vs 5 | -14.37 | -50.25 | 0.82 |

Abbreviations: AIC, Akaike information criterion; BIC, Bayesian information criterion.

**Supplementary Table 9.** Results of Model 3 (n=655, DoF=590, residual standard error =0.61). Significant effects (p<0.05) are shown in bold. The adjusted p-value with Bonferroni corrections for this non-winning model would be p<0.0008.

| Predictors | Standard β | Standard Error | Confidence Intervals | p |
| --- | --- | --- | --- | --- |
| LVF1 | -0.01 | 0.04 | -0.08 – 0.07 | 0.83 |
| LVF2 | 0.04 | 0.05 | -0.05 – 0.13 | 0.41 |
| LVF3 | -0.03 | 0.04 | -0.12 – 0.05 | 0.43 |
| Age | 0.64 | 0.05 | 0.54 – 0.75 | **<0.001** |
| Age^2^ | -0.01 | 0.04 | -0.10 – 0.07 | 0.81 |
| LVF1.Age | -0.03 | 0.04 | -0.11 – 0.05 | 0.45 |
| LVF1.Age^2^ | -0.05 | 0.04 | -0.12 – 0.03 | 0.22 |
| LVF2.Age | 0.05 | 0.05 | -0.04 – 0.14 | 0.27 |
| LVF2.Age^2^ | 0.08 | 0.04 | -0.01 – 0.16 | 0.08 |
| LVF3.Age | -0.05 | 0.04 | -0.13 – 0.04 | 0.28 |
| LVF3.Age^2^ | -0.02 | 0.04 | -0.10 – 0.06 | 0.64 |
| Anti-Hypertensives | 1.22 | 0.75 | -0.25 – 2.69 | 0.10 |
| Beta Blockers | 1.88 | 2.34 | -2.72 – 6.48 | 0.42 |
| Diuretics | -2.27 | 2.55 | -7.28 – 2.75 | 0.38 |
| Dyslipidemics | -0.66 | 0.5 | -1.64 – 0.32 | 0.19 |
| LVF1.Anti-Hypertensives | -1.46 | 1.28 | -3.98 – 1.06 | 0.26 |
| LVF1.Betablockers | -6.97 | 10.46 | -27.51 – 13.57 | 0.51 |
| LVF1.Diuretics | 7.00 | 3.16 | 0.79 – 13.20 | **0.03** |
| LVF1.Dyslipidemics | -0.83 | 0.52 | -1.85 – 0.20 | 0.11 |
| LVF2.Anti-Hypertensives | -1.04 | 0.69 | -2.40 – 0.31 | 0.13 |
| LVF2.Betablockers | 3.18 | 5.92 | -8.45 – 14.81 | 0.59 |
| LVF2.Diuretics | -3.32 | 3.92 | -11.02 – 4.39 | 0.40 |
| LVF2.Dyslipidemics | 1.16 | 0.61 | -0.05 – 2.37 | 0.06 |
| LVF3.Anti-Hypertensives | 0.29 | 1.01 | -1.69 – 2.28 | 0.77 |
| LVF3.Betablockers | -4.13 | 5.35 | -14.64 – 6.37 | 0.44 |
| LVF3.Diuretics | -0.7 | 2.87 | -6.33 – 4.94 | 0.81 |
| LVF3.Dyslipidemics | -0.66 | 1.15 | -2.91 – 1.59 | 0.57 |
| LVF1.Anti-Hypertensives.Age | 1.11 | 1.5 | -1.83 – 4.06 | 0.46 |
| LVF1.Anti-Hypertensives.Age^2^ | -0.03 | 0.73 | -1.46 – 1.40 | 0.97 |
| LVF1.Betablockers.Age | 6.41 | 9.93 | -13.09 – 25.92 | 0.52 |
| LVF1.Betablockers.Age^2^ | -1.21 | 3 | -7.10 – 4.69 | 0.69 |
| LVF1.Diuretics.Age | -7.05 | 3.41 | -13.75 – -0.35 | 0.04 |
| LVF1.Diuretics.Age^2^ | 2.39 | 1.31 | -0.18 – 4.95 | 0.07 |
| LVF1.Dyslipidemics.Age | 1.07 | 0.6 | -0.12 – 2.26 | 0.08 |
| LVF1.Dyslipidemics.Age^2^ | -0.73 | 0.42 | -1.55 – 0.10 | 0.08 |
| LVF2.Anti-Hypertensives.Age | 1.25 | 0.84 | -0.40 – 2.89 | 0.14 |
| LVF2.Anti-Hypertensives.Age^2^ | -0.92 | 0.48 | -1.87 – 0.02 | 0.06 |
| LVF2.Betablockers.Age | -3.56 | 5.53 | -14.43 – 7.30 | 0.52 |
| LVF2.Betablockers.Age^2^ | 0.75 | 1.67 | -2.53 – 4.03 | 0.65 |
| LVF2.Diuretics.Age | 3.85 | 4.32 | -4.62 – 12.33 | 0.37 |
| LVF2.Diuretics.Age^2^ | -1.38 | 1.87 | -5.04 – 2.29 | 0.46 |
| LVF2.Dyslipidemics.Age | -1.45 | 0.68 | -2.79 – -0.12 | **0.03** |
| LVF2.Dyslipidemics.Age^2^ | 1.13 | 0.36 | 0.41 – 1.84 | **<0.01** |
| LVF3.Anti-Hypertensives.Age | -0.06 | 1.16 | -2.34 – 2.22 | 0.96 |
| LVF3.Anti-Hypertensives.Age^2^ | 0.08 | 0.6 | -1.10 – 1.27 | 0.89 |
| LVF3.Betablockers.Age | 5.09 | 5.7 | -6.10 – 16.27 | 0.37 |
| LVF3.Betablockers.Age^2^ | -2.77 | 2.52 | -7.71 – 2.17 | 0.27 |
| LVF3.Diuretics.Age | 1.12 | 3.09 | -4.95 – 7.19 | 0.72 |
| LVF3.Diuretics.Age^2^ | -0.65 | 1.42 | -3.43 – 2.14 | 0.65 |
| LVF3.Dyslipidemics.Age | 0.62 | 1.27 | -1.87 – 3.12 | 0.62 |
| LVF3.Dyslipidemics.Age^2^ | -0.25 | 0.63 | -1.49 – 0.99 | 0.69 |
| Anti-Hypertensives.Age | -1.07 | 0.91 | -2.85 – 0.71 | 0.24 |
| Anti-Hypertensives.Age^2^ | 0.68 | 0.53 | -0.36 – 1.72 | 0.20 |
| Beta Blockers.Age | -0.86 | 2.44 | -5.66 – 3.94 | 0.72 |
| Beta Blockers.Age^2^ | 0.15 | 1.21 | -2.22 – 2.53 | 0.90 |
| Diuretics.Age | 2.24 | 2.87 | -3.40 – 7.88 | 0.44 |
| Diuretics.Age^2^ | -1.01 | 1.34 | -3.63 – 1.62 | 0.45 |
| Dyslipidemics.Age | 0.81 | 0.57 | -0.31 – 1.92 | 0.16 |
| Dyslipidemics.Age^2^ | -0.66 | 0.4 | -1.46 – 0.13 | 0.10 |
| Sex | 0.04 | 0.06 | -0.08 – 0.16 | 0.46 |
| Education |  |  |  |  |
| No qualifications tried | -0.75 | 0.52 | -1.77 – 0.27 | 0.15 |
| GCSEs / O-levels | -0.81 | 0.51 | -1.81 – 0.19 | 0.11 |
| A-levels | -0.53 | 0.51 | -1.52 – 0.47 | 0.30 |
| Degree | -0.42 | 0.5 | -1.41 – 0.57 | 0.40 |

Abbreviations: LVF1-3, latent vascular factors.

**Supplementary Table 10.** Results of Model 4 (n=655, DoF=627, residual standard error =0.65). Significant effects (p<0.05) are shown in bold. The adjusted p-value with Bonferroni corrections for this non-winning model would be p<0.002.

| Predictors | Standard β | Standard Error | Confidence Intervals | p |
| --- | --- | --- | --- | --- |
| LVF1 | -0.02 | 0.12 | -0.25 – 0.21 | 0.85 |
| LVF2 | -0.01 | 0.13 | -0.26 – 0.23 | 0.93 |
| LVF3 | 0.08 | 0.13 | -0.17 – 0.34 | 0.52 |
| Age | 0.58 | 0.15 | 0.29 – 0.87 | **<0.001** |
| Age^2^ | 0.05 | 0.13 | -0.20 – 0.29 | 0.72 |
| LVF1.Age | -0.12 | 0.12 | -0.35 – 0.10 | 0.29 |
| LVF1.Age^2^ | 0.06 | 0.11 | -0.15 – 0.28 | 0.55 |
| LVF2.Age | 0.04 | 0.13 | -0.22 – 0.31 | 0.74 |
| LVF2.Age^2^ | 0.04 | 0.12 | -0.20 – 0.28 | 0.73 |
| LVF3.Age | 0.08 | 0.13 | -0.17 – 0.34 | 0.52 |
| LVF3.Age^2^ | 0.09 | 0.12 | -0.14 – 0.33 | 0.44 |
| Sex | -0.06 | 0.08 | -0.22 – 0.11 | 0.50 |
| LVF1.Sex | 0.01 | 0.07 | -0.13 – 0.15 | 0.88 |
| LVF2.Sex | -0.01 | 0.08 | -0.16 – 0.15 | 0.92 |
| LVF3.Sex | -0.04 | 0.08 | -0.20 – 0.11 | 0.59 |
| Age.Sex | 0.10 | 0.09 | -0.09 – 0.28 | 0.30 |
| Age^2^.Sex | -0.02 | 0.08 | -0.17 – 0.14 | 0.83 |
| LVF1.Age.Sex | 0.08 | 0.07 | -0.06 – 0.22 | 0.29 |
| LVF1.Age^2^.Sex | -0.06 | 0.07 | -0.20 – 0.07 | 0.37 |
| LVF2.Age.Sex | -0.01 | 0.08 | -0.18 – 0.15 | 0.89 |
| LVF2.Age^2^.Sex | 0.02 | 0.08 | -0.13 – 0.17 | 0.77 |
| LVF3.Age.Sex | -0.05 | 0.08 | -0.20 – 0.11 | 0.56 |
| LVF3.Age^2^.Sex | -0.05 | 0.07 | -0.19 – 0.09 | 0.50 |
| Education |  |  |  |  |
| No qualifications tried | -0.58 | 0.53 | -1.63 – 0.47 | 0.28 |
| GCSEs / O-levels | -0.66 | 0.53 | -1.69 – 0.38 | 0.21 |
| A-levels | -0.44 | 0.52 | -1.47 – 0.59 | 0.40 |
| Degree | -0.31 | 0.52 | -1.33 – 0.72 | 0.56 |

Abbreviations: LVF1-3, latent vascular factors.

**Supplementary Table 11.** Results of Model 5 (n=655, DoF=626, residual standard error =0.64). Significant effects (p<0.05) are shown in bold. The adjusted p-value with Bonferroni corrections for this non-winning model would be p<0.002.

| Predictors | Standard β | Standard Error | Confidence Intervals | p |
| --- | --- | --- | --- | --- |
| LVF1 | 0.01 | 0.05 | -0.08 – 0.10 | 0.83 |
| LVF2 | 0.01 | 0.05 | -0.09 – 0.11 | 0.90 |
| LVF3 | 0.01 | 0.05 | -0.08 – 0.11 | 0.80 |
| Age | 0.71 | 0.05 | 0.62 – 0.81 | **<0.001** |
| Age^2^ | 0.02 | 0.04 | -0.07 – 0.10 | 0.66 |
| LVF1.Age | -0.01 | 0.05 | -0.11 – 0.09 | 0.90 |
| LVF1.Age^2^ | -0.03 | 0.04 | -0.11 – 0.05 | 0.49 |
| LVF2.Age | 0.01 | 0.06 | -0.10 – 0.12 | 0.91 |
| LVF2.Age^2^ | 0.09 | 0.04 | 0.01 – 0.18 | **0.04** |
| LVF3.Age | -0.01 | 0.05 | -0.10 – 0.09 | 0.88 |
| LVF3.Age^2^ | 0.01 | 0.04 | -0.08 – 0.09 | 0.85 |
| LVF1.LVF2 | 0.01 | 0.05 | -0.08 – 0.10 | 0.81 |
| LVF1.LVF3 | 0.03 | 0.04 | -0.06 – 0.11 | 0.54 |
| LVF2.LVF3 | <0.01 | 0.06 | -0.11 – 0.11 | 0.96 |
| LVF1.LVF2.LVF3 | 0.03 | 0.04 | -0.06 – 0.11 | 0.56 |
| LVF1.LVF2.Age | -0.04 | 0.05 | -0.12 – 0.05 | 0.44 |
| LVF1.LVF2.Age^2^ | <0.01 | 0.03 | -0.06 – 0.06 | 1.00 |
| LVF1.LVF3.Age | -0.04 | 0.05 | -0.13 – 0.05 | 0.39 |
| LVF1.LVF3.Age^2^ | -0.05 | 0.04 | -0.13 – 0.03 | 0.26 |
| LVF2.LVF3.Age | 0.02 | 0.06 | -0.10 – 0.14 | 0.78 |
| LVF2.LVF3.Age^2^ | 0.01 | 0.05 | -0.08 – 0.11 | 0.78 |
| LVF1.LVF2.LVF3.Age | -0.01 | 0.05 | -0.10 – 0.09 | 0.92 |
| LVF1.LVF2.LVF3.Age^2^ | -0.01 | 0.03 | -0.07 – 0.05 | 0.76 |
| Sex | <0.01 | 0.06 | -0.12 – 0.12 | 0.99 |
| Education |  |  |  |  |
| No qualifications tried | -0.62 | 0.53 | -1.65 – 0.42 | 0.25 |
| GCSEs / O-levels | -0.7 | 0.52 | -1.73 – 0.32 | 0.18 |
| A-levels | -0.48 | 0.52 | -1.50 – 0.54 | 0.36 |
| Degree | -0.35 | 0.52 | -1.37 – 0.66 | 0.49 |

Abbreviations: LVF1-3, latent vascular factors.

SUPPLEMENTARY MATERIAL: BIBLIOGRAPHY

1. Shafto, M. A. *et al.* The Cambridge Centre for Ageing and Neuroscience (Cam-CAN) study protocol: a cross-sectional, lifespan, multidisciplinary examination of healthy cognitive ageing. *BMC Neurol.* **14**, 204 (2014).

2. Asparouhov, T. & Muthén, B. Exploratory Structural Equation Modeling. *Struct. Equ. Model. Multidiscip. J.* **16**, 397–438 (2009).

3. Chen, W. *et al.* Age-related patterns of the clustering of cardiovascular risk variables of syndrome X from childhood to young adulthood in a population made up of black and white subjects: the Bogalusa Heart Study. *Diabetes* **49**, 1042–1048 (2000).

4. Goodman, E., Dolan, L. M., Morrison, J. A. & Daniels, S. R. Factor analysis of clustered cardiovascular risks in adolescence: obesity is the predominant correlate of risk among youth. *Circulation* **111**, 1970–1977 (2005).

5. Khader, Y. S. *et al.* Factor analysis of cardiometabolic risk factors clustering in children and adolescents. *Metab. Syndr. Relat. Disord.* **9**, 151–156 (2011).

6. Mayer-Davis, E. J. *et al.* Cardiovascular disease risk factors in youth with type 1 and type 2 diabetes: implications of a factor analysis of clustering. *Metab. Syndr. Relat. Disord.* **7**, 89–95 (2009).

7. Tsvetanov, K. A. *et al.* The effects of age on resting-state BOLD signal variability is explained by cardiovascular and cerebrovascular factors. *Psychophysiology* **58**, e13714 (2021).

8. Borgeest, G. S. *et al.* Greater lifestyle engagement is associated with better age-adjusted cognitive abilities. *PLOS ONE* **15**, e0230077 (2020).

9. Cattell, R. B. The measurement of adult intelligence. *Psychol. Bull.* **40**, 153–193 (1943).

10. McDonough, I. M. *et al.* Discrepancies between Fluid and Crystallized Ability in Healthy Adults: A Behavioral Marker of Preclinical Alzheimer’s Disease. *Neurobiol. Aging* **46**, 68–75 (2016).

11. Revelle, W. psych: Procedures for Personality and Psychological Research, Northwestern University, Evanston, Illinois, USA, https://CRAN.R-project.org/package=psych. (2017).

12. Rosseel, Y. lavaan: An R Package for Structural Equation Modeling. *J. Stat. Softw.* **48**, 1–36 (2012).

13. Kolbe, L., Molenaar, D., Jak, S. & Jorgensen, T. D. Assessing measurement invariance with moderated nonlinear factor analysis using the R package OpenMx. *Psychol. Methods* (2022) doi:10.1037/met0000501.

14. Recommendations | Hypertension in adults: diagnosis and management | Guidance | NICE. https://www.nice.org.uk/guidance/ng136/chapter/Recommendations#diagnosing-hypertension.
